# NatB regulates Rb mutant cell death and tumor growth by modulating EGFR/MAPK signaling through the N-end rule pathways

**DOI:** 10.1371/journal.pgen.1008863

**Published:** 2020-06-19

**Authors:** Zhentao Sheng, Wei Du

**Affiliations:** Ben May Department for Cancer Research, The University of Chicago, Chicago, Illinois, United States of America; Harvard Medical School, Howard Hughes Medical Institute, UNITED STATES

## Abstract

Inactivation of the Rb tumor suppressor causes context-dependent increases in cell proliferation or cell death. In a genetic screen for factors that promoted Rb mutant cell death in *Drosophila*, we identified Psid, a regulatory subunit of N-terminal acetyltransferase B (NatB). We showed that NatB subunits were required for elevated EGFR/MAPK signaling and Rb mutant cell survival. We showed that NatB regulates the posttranscriptional levels of the highly conserved pathway components Grb2/Drk, MAPK, and PP2AC but not that of the less conserved Sprouty. Interestingly, NatB increased the levels of positive pathway components Grb2/Drk and MAPK while decreased the levels of negative pathway component PP2AC, which were mediated by the distinct N-end rule branch E3 ubiquitin ligases Ubr4 and Cnot4, respectively. These results suggest a novel mechanism by which NatB and N-end rule pathways modulate EGFR/MAPK signaling by inversely regulating the levels of multiple conserved positive and negative pathway components. As inactivation of Psid blocked EGFR signaling-dependent tumor growth, this study raises the possibility that NatB is potentially a novel therapeutic target for cancers dependent on deregulated EGFR/Ras signaling.

## Author summary

Nt-acetylation is often regarded as a constitutive, irreversible, and static modification that is not suited to serve regulatory functions. Our observation that Nt-acetylation by NatB coordinately regulates some of the highly conserved positive and negative components of the EGFR/MAPK pathway suggest that Nt-acetylation and N-end rule pathways can play important roles regulating important signaling pathways. As these positive and negative pathway components are conserved and shared by multiple growth factor signaling pathways and as Acetyl-CoA level, which is influenced by cell metabolism, can be rate limiting for Nt-acetylation, our results also suggest a potentially new mechanism by which cellular metabolic status can regulate growth factor signaling.

## Introduction

Rb is a tumor suppressor often inactivation in cancers [1,2]. Rb binds to large numbers of interacting proteins including E2F transcription factors and chromatin regulators to modulate diverse cellular processes, including cell proliferation, cell cycle checkpoint and DNA damage repair, chromatin dynamics, apoptosis, cell differentiation, etc [3,4,5,6,7,8]. Interestingly, the consequences of Rb inactivation in different cell types can be different, ranging from increased cell proliferation, increased cell death, or altered cell differentiation depending on specific cell context. Understanding the mechanisms that mediate the context dependent effect of Rb-inactivation, particularly in an *in vivo* system, will provide novel insights into the *in vivo* vulnerability of Rb mutant cells, which can potentially promote the development of novel therapeutic approaches to target cancers with inactivated Rb [9,10].

The Rb pathway is highly conserved and more streamlined in *Drosophila* [6,7,11,12,13,14]. Interestingly, inactivation of the fly Retinoblastoma (Rb) homolog Rbf in the developing eye discs lead to ectopic cell proliferation in posterior undifferentiated cells but increased cell death in cells just anterior to the morphogenetic furrow (MF), where the eye progenitor cells arrest in G1 and initiate photoreceptor differentiation [15,16,17]. Therefore, the biological consequences of Rbf-inactivation are different in distinct cell types, suggesting that *Drosophila* developing eye is an excellent model system to dissect the cell context-dependent modulation of Rb-inactivation *in vivo*.

Studies of the *rbf* mutant cell death just anterior to the MF revealed that *rbf*-inactivation leads to elevated Hid expression, a cell death regulator repressed by Rbf [17]. Interestingly, inactivation of *tsc2* or *axin*, in conjunction with *rbf* inactivation, promoted synergistic cell death mediated by deregulated TORC1 and E2F1 activity that induced excessive cellular stress including metabolic stress and DNA damage stress [18,19,20]. In support of the idea that metabolic alteration contributed to rbf mutant cell death, a recent single cell RNA sequencing (scRNA-seq) study of *rbf* mutant eye discs revealed that the altered metabolic activity with increased glycolysis and increased intracellular acidification contributed to the *rbf* mutant cell death near the MF [21].

In addition to metabolic alterations, *rbf* mutant cell death in the MF is also highly sensitive to the level of EGFR/MAPK signaling and elevating the EGFR/MAPK signaling inhibited these cell death [16,22]. EGFR/MAPK signaling is normally activated in the MF and in posterior eye disc regions and plays critical roles in preventing cell death in addition to its role in regulating cell cycle and differentiation [23]. Interestingly, reducing EGFR signaling by mutations that function upstream of MAPK preferentially increased apoptosis of *rbf* mutant cells in posterior eye discs [16,24]. In contrast, mutation of *rno*, which functions downstream of MAPK and regulates the nuclear output of EGFR signaling [25], did not affect apoptosis of *rbf* mutant cells. These results are consistent with the reported direct regulation of Hid by MAPK [26]. Taken together, these results suggest that *rbf* mutant cells are more sensitive than WT cells to reduced level of EGFR/MAPK signaling and that the *rbf* synthetic lethal screens [18,19,24] can identify additional modulators of EGFR signaling. In this study, we report new alleles of NatB subunit in *rbf* synthetic lethal screen and identify a novel mechanism by which NatB modulates EGFR signaling by coordinately regulating the levels of positive and negative components through the two branches of the N-end rule pathway.

## Results

### Inactivation of NatB subunits induced synergistic cell death with loss of *rbf* in developing *Drosophila* tissues

**Fig 1:**
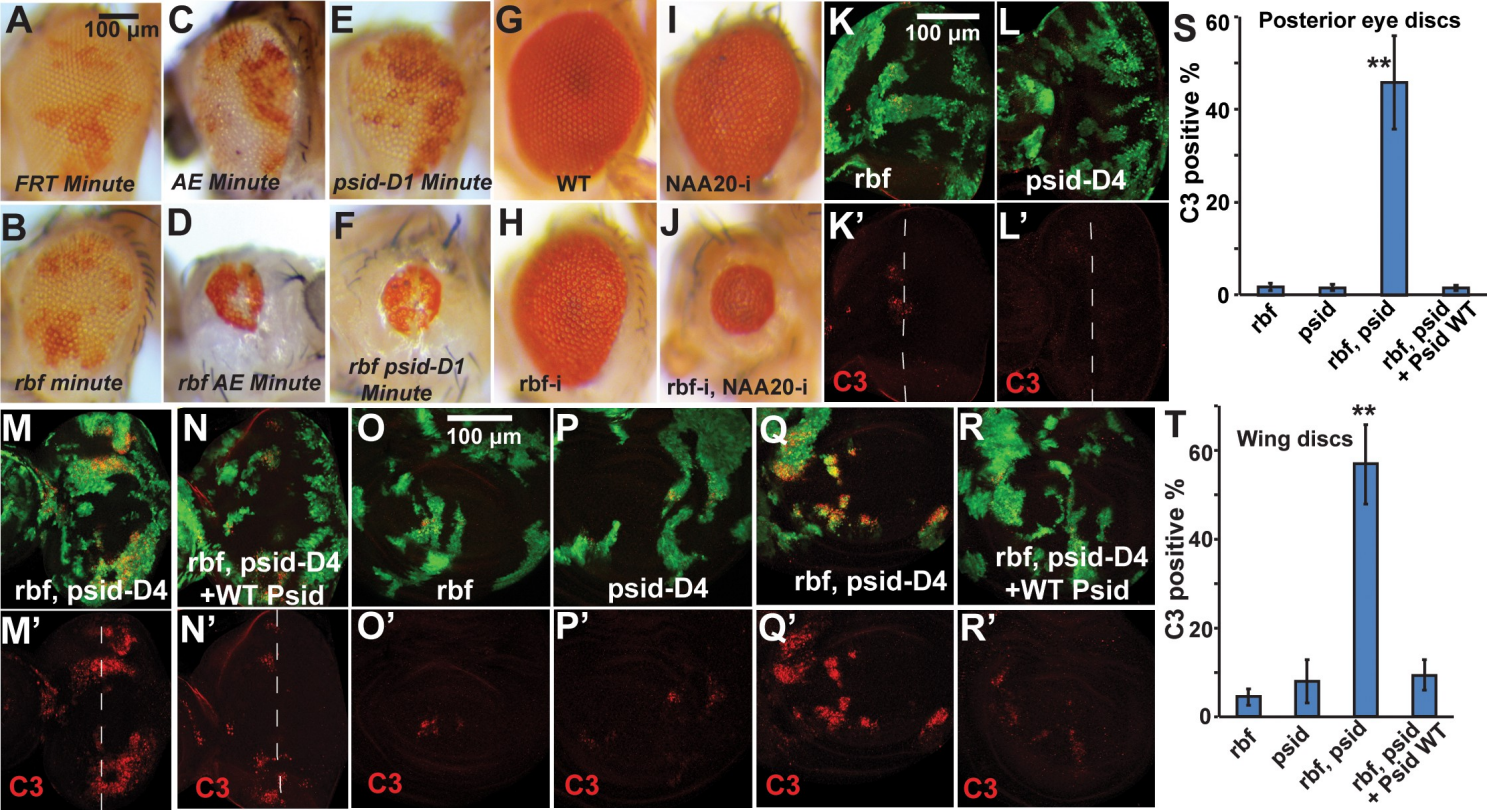
Inactivation of NatB subunits, Psid or NAA20, induced synergistic cell death with loss of *rbf* in *Drosophila*. (A-F) Adult eyes with clones in *Minute* background. WT control (A), *rbf*, *psidin* single or double mutation (B-F). *AE* and *psid-D1* (*psid*^*85D1*^) are two different *psidin* alleles. Mutant clones are marked by lack of red pigment. Large clones (white patches) of *psidin* or *rbf* single mutant clones (B, C, and E) were observed in the *Minute* background. In contrast, *rbf psidin* double mutant clones generated in the *Minute* background were mostly lost, resulting in smaller eyes (D and F). (G-J) Adult eyes with single or double RNAi of Rbf or NAA20 were shown. (K-T) Levels of C3 levels in 3^rd^ instar eye discs (K-N’) or wing discs (O-R’) with *rbf*, or *psidin*, or *rbf psidin* double mutant clones are shown. Mutant clones were marked by GFP expression and cleaved caspase 3 staining was shown in red. Dash line indicates the position of furrow region in developing eye discs (K-N). Levels of C3 staining in mutant clones located in posterior eye discs and wing discs were quantified and the average and standard deviation were shown in (S) and (T), respectively (N≥4 for each genotype). In both posterior eye discs (K-M, S) and wing discs (O-Q, T), the levels of C3 staining were significantly increased in *rbf*, *psidin* double mutant clones in comparison to the single mutant clones. Expression of WT Psid rescued the C3 levels in *rbf*, *psidin* double mutant clones in both eye (M-N, S) and wing discs (Q-R, T). ** indicates statistically significant differences (p<0.00001, t test) between double and single mutant clones or between double mutant clones with or without WT Psid rescue. The black or white bars indicate 100 μM. In this and all the subsequent figures, discs are orientated posterior to the right, genotypes of mutant clones and anti-staining are indicated on the figure.

In a genetic screen to identify *rbf* synthetic lethal mutations on *Drosophila* chromosome 3R, we identified four mutants (*AB*, *AE*, *CJ*, and *EQ*) that fall into the same complementation group. While clones of FRT control or single mutant (paled patches) were readily detected in adult eyes, double mutant clones of *rbf* and these mutants were not detectable (S1A–S1F Fig). Because *Minute* mutant cells have growth disadvantage and will be outcompeted and eliminated by the surrounding WT cells [27], mutant clones generated in the *Minute* background will allow the development of adult eyes that consist of mostly the mutant cells. Therefore, we generated *rbf* and *AE* group single or double mutant clones in the *Minute* background to better visualize the loss of double mutant tissues. Indeed, large white patches of *rbf*, *AE*, *EQ*, *AB*, and *CJ* single mutant clones were observed in *Minute* background (Fig 1A–1C and S1K, S1M and S1O Fig). Interestingly, most of the white patches were lost in conjunction with *rbf* mutation, leading to the development of much smaller adult eyes (Fig 1D and S1L, S1N and S1P Fig). These results strongly support the notion that the identified mutants induce synthetic lethality in conjunction with *rbf* mutation.

Genetic mapping using 3R deficiencies was carried out. We found that the *AE* complementation group mutants map to the 92B4-C1 cytological region. The following results demonstrate that the *AE* complementary group mutants are alleles of *psid*. First, both of the two existing *psid* alleles, *psid-D4* (*psid*^*55D4*^) and *psid-D1* (*psid*^*85D1*^) [28,29], failed to complement each of the *AE* group mutants; Second, both *psid-D4* and *psid-D1* showed loss of mutant clones in the presence of *rbf* mutation, similar to that of *AE* and *EQ* mutants (Fig 1E and 1F, S1G–S1J Fig). Third, sequencing revealed that *AE* and *CJ* mutants contained stop codon mutations in Psid that changed amino acid Trp^264^ (TGG) and Gln^814^ (CAG) to stop codon (TAG), respectively.

To demonstrate that *psid* mutation induced synthetic lethality with *rbf*, we carried out immunostaining of the developing discs using the anti cleaved caspase 3 antibody (C3). *rbf* mutant cells showed significantly increased cell death just anterior to the morphogenetic furrow (MF) but not much in the posterior region of the developing eye disc [15,17] (Fig 1K and 1K’). While *psid* single mutant clones did not increase C3 level in posterior eye discs (Fig 1L and 1L’), significant increased C3 level was observed in *rbf*, *psid* double mutant clones in posterior eye discs (Fig 1M and 1M’ and 1S). Importantly, expression of WT Psid rescued the observed synergistically increased C3 level in posterior eye disc (Fig 1N and 1N’ and 1S), indicating that the increased cell death depended on the loss of Psid function. Furthermore, synergistically increased C3 level of the *rbf*, *psid* double mutant cells, which can be rescued by expression of WT Psid, was also observed in the wing discs (Fig 1O–1R’ and 1T), indicating that the synergistic cell death effects of *rbf psid* double mutants are not limited to the eye discs.

Psid, which encodes the regulatory subunit of NatB, can function through two distinct mechanisms. Psid was shown to affect cell migration and axon guidance of olfactory receptor neurons by binding to actin and antagonizing tropomyosin [28,29]. In addition, Psid was also required for olfactory receptor neuron survival by binding to the NatB catalytic subunit NAA20 [29]. NatB catalyzes the addition of acetyl group to the N terminus of proteins that start with MD, ME, MN, or MQ [30]. To determine whether the observed interaction between *psid* and *rbf* is mediated through its interaction with NAA20, we tested the effects of knockdown NAA20. Knockdown of NAA20 by RNAi in conjunction with *Rbf* RNAi also led to significantly decreased adult eye sizes (Fig 1G–1J), which was correlated with significantly increased C3 levels in eye/antenna discs (S1Q–S1S Fig). Taken together, these data suggest that inactivation of NatB induced synergistic cell death with loss of *rbf*.

### NatB regulates endogenous EGFR/MAPK signaling in the developing eye disc

**Fig 2:**
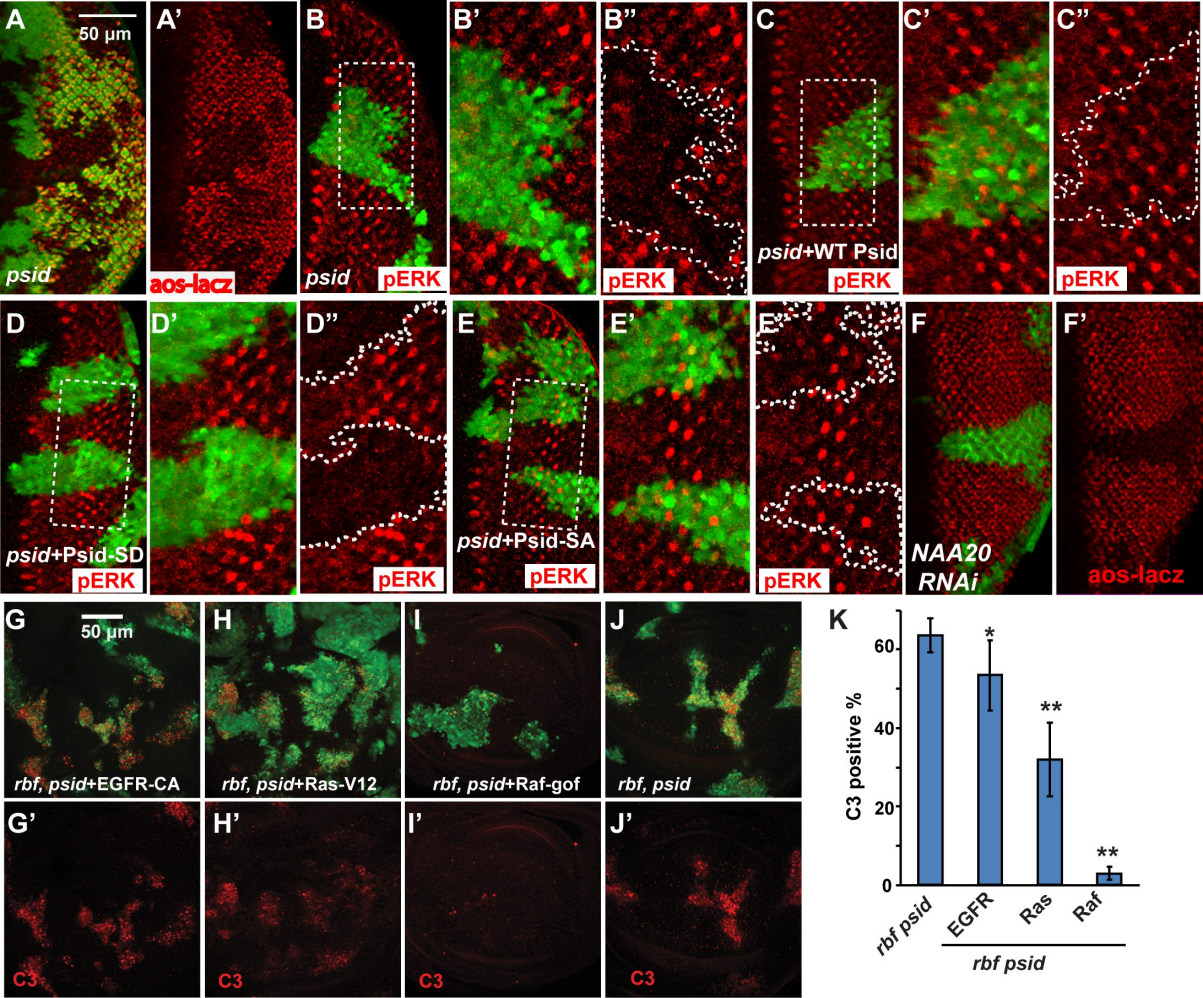
NatB was required for the elevated EGFR signaling in posterior eye discs. (A and A’) *psid-D4* clones in posterior of eye discs were marked by the absence of GFP. The level of EGFR signaling, as shown by the expression of aos-lacZ reporter detected by anti β-galactosidase staining (red), was significantly decreased in *psid-D4* clones. (B-E") MARCM clones of *psid-D4* in the posterior of eye discs were marked by GFP expression. EGFR signaling, as shown by the level of pERK, was significantly decreased in *psid-D4* clone (B-B"), and was restored by expressing WT Psid (C-C") or the nonphosphorylatable Psid^SA^ (S678A) (E-E") but not the phosphomimetic Psid^SD^ (S678D) mutant (D-D"). (F and F’) NAA20 RNAi clones marked by GFP expression also decreased EGFR signaling as shown by decreased expression level of aos-lacZ. (G-K) Cell death in *rbf*, *psidin* double mutant clones with different UAS-transgene expression in wing discs were determined by C3 staining (red). The double mutant clones were marked by GFP expression. While activated EGFR (G) did not inhibit cell death observed in *rbf*, *psid* double mutant clones (J), activated Ras partially inhibited cell death (H), and activated Raf inhibited cell death to background levels (I). (K) The levels of C3 staining in mutant clones located in wing discs were quantified and the average and standard deviation were shown. The statistically significant difference between triple and double mutant clones were showed (N≥7 for each genotype; ** indicates P<0.000001, * indicates P = 0.01, t test).

Our previous studies suggest that mutations such as *TSC2* and *axin* induce synergistic cell death with *rbf* most strongly in anterior eye discs due to induction of excessive cellular stress [18,19,20]. In contrast, mutations that cause deficiency in EGFR/MAPK signaling induce synergistic cell death with *rbf* mainly in posterior eye discs [24]. The observed *psid*, *rbf* synergistic cell death in posterior eye discs prompted us to determine whether *psid* mutation affect EGFR/MAPK signaling. EGFR signaling is upregulated in posterior eye discs, which play important roles regulating cell cycle, cell survival, and differentiation [23,31]. Indeed, *psid* mutation significantly decreased EGFR signaling as shown by reduced Aos-lacZ reporter expression and reduced level of active Diphosphorylated ERK (pERK) in *psid* mutant clones (Fig 2A and 2B”, S2A–S2C’ Fig). Furthermore, expression of WT Psid restored the pERK levels (Fig 2C and 2C”). Interestingly, expression of the phosphomimetic Psid mutant (Psid^S678D^), which mutated a conserved Ser that was shown to be phosphorylated in human MDM20 [32], failed to rescue (Fig 2D and 2D”). In contrast, expression of the nonphosphorylatable Psidin^S687A^ mutant did rescue (Fig 2E and 2E”). As the Psid^S678D^ mutant is defective in binding to NAA20 while the Psid^S687A^ mutant retains the ability to bind NAA20 [29], these results suggest that the reduced MAPK activation in *psid* mutant clones is mediated by reduced NatB activity. In support of this, knockdown of NAA20 in eye discs also significantly reduced EGFR signaling as shown by reduced Aos-lacZ reporter expression (Fig 2F and 2F’) and reduced pERK levels (S2D and S2D’ Fig).

EGFR signaling ligand in eye disc was released by the developing photoreceptor cells, particularly the R8 photoreceptor cells in the developing eye discs [33]. Knockdown of NAA20 slightly delayed the formation of R8 equivalence groups and the differentiation of R8 and additional photoreceptor cells (S2E–S2F’ Fig). However, the differentiation of photoreceptor cells was not blocked in the posterior NatB-inactivated cells where decreased EGFR signaling was observed. These results suggest that the observed inhibition of EGFR signaling by *psid* mutation is not simply due to an absence of EGFR ligand caused by inhibition of photoreceptor differentiation.

### NatB regulates MAPK activation at multiple points downstream of EGFR

**Fig 3:**
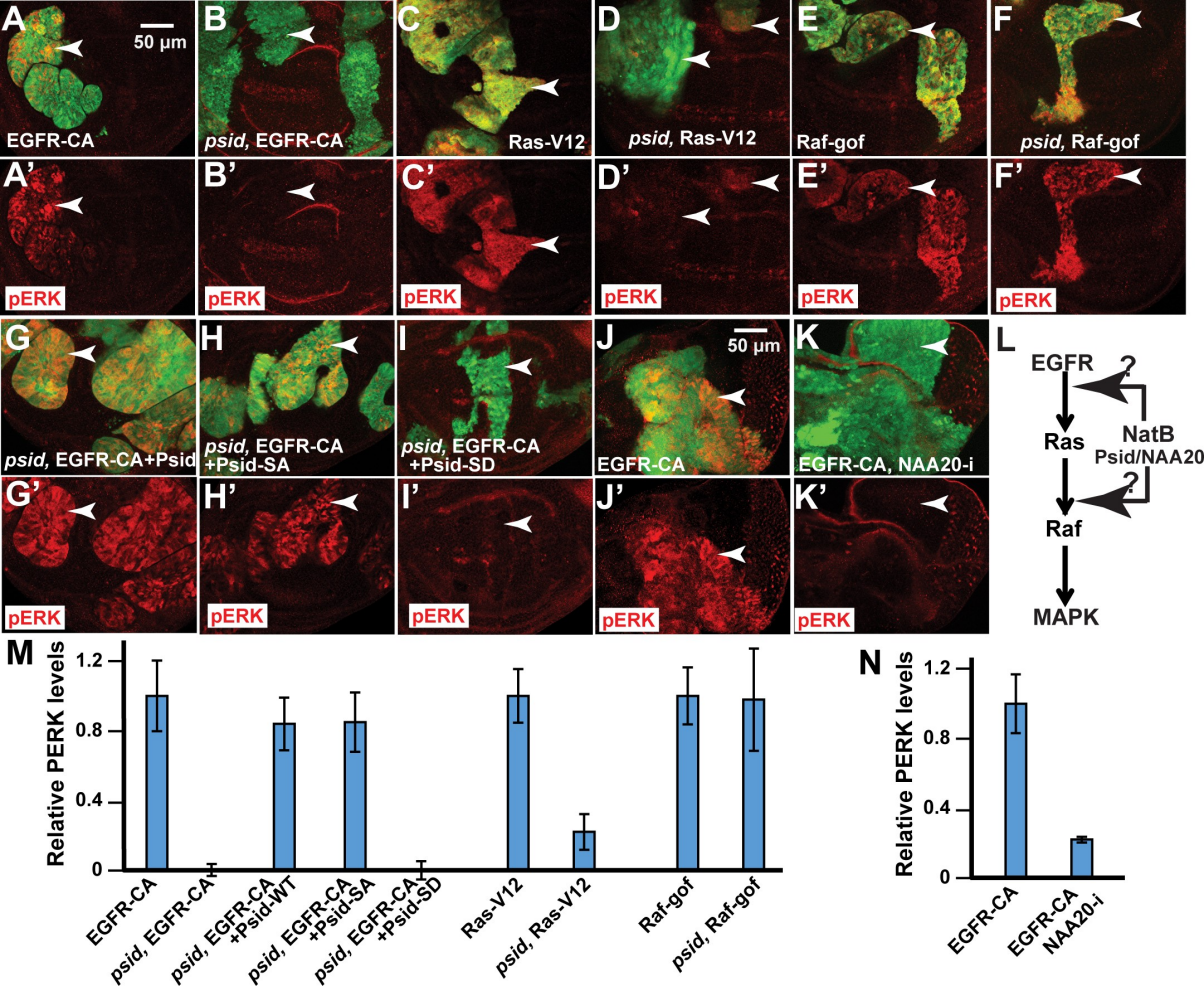
Inactivation of Nat B subunits inhibited MAPK activation at multiple points downstream of EGFR. (A-F') The effects of *psidin* (*psid-D4*) mutation on the MAPK activation (pERK staining, red) induced by expressing activated EGFR, Ras, or Raf in the wing discs are shown. *psid-D4* mutant clones with activated EGFR, Ras, or Raf expression were marked by GFP expression (pointed by arrowheads). Activated EGFR-induced pERK was completely inhibited by *psid-D4* mutation (A-B’). In contrast, activated Ras-induced pERK was partially inhibited by *psid-D4* mutation (C-D’) while activated Raf-induced pERK was not significantly inhibited (E-F’). (G-I’) Inhibition of activated EGFR-induced pERK in *psid* (*psid-D4*) mutant clones was rescued by expressing WT Psid (G) or the nonphosphorylatable Psid^SA^ (S678A) (H) but not the phosphomimetic mutant Psid^SD^ (S678D) (I). (J-K') Using EyeFlp-CoinGal4, activated EGFR-induced pERK (J) was largely inhibited by NAA20 RNAi in eye disc (K). (L) A diagram that summarizes the main points that NatB regulated EGFR/MAPK signaling is shown. (M-N) Relative pERK levels in different genotype clones in (A) to (K) were quantified and the average and standard deviation were shown in (M) and (N), respectively (N≥4 for each genotype).

To further investigate how *psid* mutation inhibits EGFR signaling, we determined the effect of *psid* mutation on MAPK activation induced by expressing activated EGFR, Ras, or Raf in *psid* mutant or WT control cells using MARCM approach. Interestingly, activated EGFR significantly increased pERK levels in WT control but not in *psid* mutant clones (Fig 3A, 3B’ and 3M). In contrast, active Raf-induced pERK levels were not obviously inhibited by *psid* mutation (Fig 3E, 3F’ and 3M, S3C–S3D’ Fig). On the other hand, even though activated Ras-induced pERK was largely inhibited by *psid* mutation, slightly elevated pERK levels can still be observed in *psid* mutants (Fig 3C and 3D’ and 3M, S3A–S3B’ Fig). These results suggest that *psid* mutation blocks MAPK activation at multiple points downstream of activated EGFR and upstream of activated Raf (Fig 3L). To demonstrate that the observed inhibition of activated EGFR-induced pERK level is mediated by the loss of NatB activity, the ability of expressing WT or mutant Psid proteins to rescue EGFR-induced pERK was determined. The WT and S687A mutant form of Psid that can bind NAA20 were able to restore EGFR-induced pERK levels in *psid* mutant clones (Fig 3G-3H’ and 3M) while the NAA20 binding defective Psid^S678D^ mutant failed to rescue (Fig 3I-3I’ and 3M). Furthermore, knockdown of NAA20 by RNAi also strongly inhibited activated EGFR-induced pERK (Fig 3J-3K’ and 3N). Taken together, these results suggest that NatB modulates the activities of multiple targets that function between EGFR and Raf to regulate MAPK activation (Fig 3L).

To determine whether inhibition of EGFR signaling induced MAPK activation contributed to the synergistic cell death of *rbf*, *psid* double mutant cells, we tested the ability of activated EGFR, Ras, or Raf to rescue *rbf*, *psid* double mutant cell death. While activated EGFR marginally affected *rbf*, *psid* cell death, activated Ras induced significant rescue and activated Raf rescued cell death to background levels (Fig 2G–2K). These results, which correlated with the observed effects of *psid* mutation on EGFR signaling (Fig 3A–3N), support the notion that reduced MAPK activity in *psid* mutant clones contributed to the synergistic cell death with *rbf*.

### NatB stabilizes Drk, which promotes EGFR induced MAPK activation

Nt-acetylation, which involves the transfer of an acetyl group from acetyl-CoA to the α-amino group of a protein, is observed in majority of the proteins in eukaryotes. NatB is one of the six N-terminal acetyltransferases, NatA-F, in higher eukaryotes that catalyze Nt-acetylation with different substrate specificity [34]. Nt-acetylation neutralizes the positive charge and alters chemical properties of the N terminus of the protein, which has been shown to affect localization, interaction, and/or degradation of specific proteins [35]. Since NatB catalyze the addition of acetyl group to the N terminal proteins that start with MD, ME, MN, or MQ, we analyzed components of the EGFR/MAPK pathway and identified six potential NatB targets that function between EGFR and MAPK: Drk (fly Grb2 homolog), PP2AC (catalytic subunit of PP2A), Sprouty (Spry), PTP-ER, Sur-8, and MAPK. Two of these proteins, Drk and PP2AC, showed high sequence conservation in the first two amino acids, the N-terminal region, as well as the overall protein. The high levels of sequence conservation suggest that the regulation of these two proteins by NatB and the overall function of these proteins are likely conserved. Therefore, we initially focused on Drk and PP2AC.

**Fig 4:**
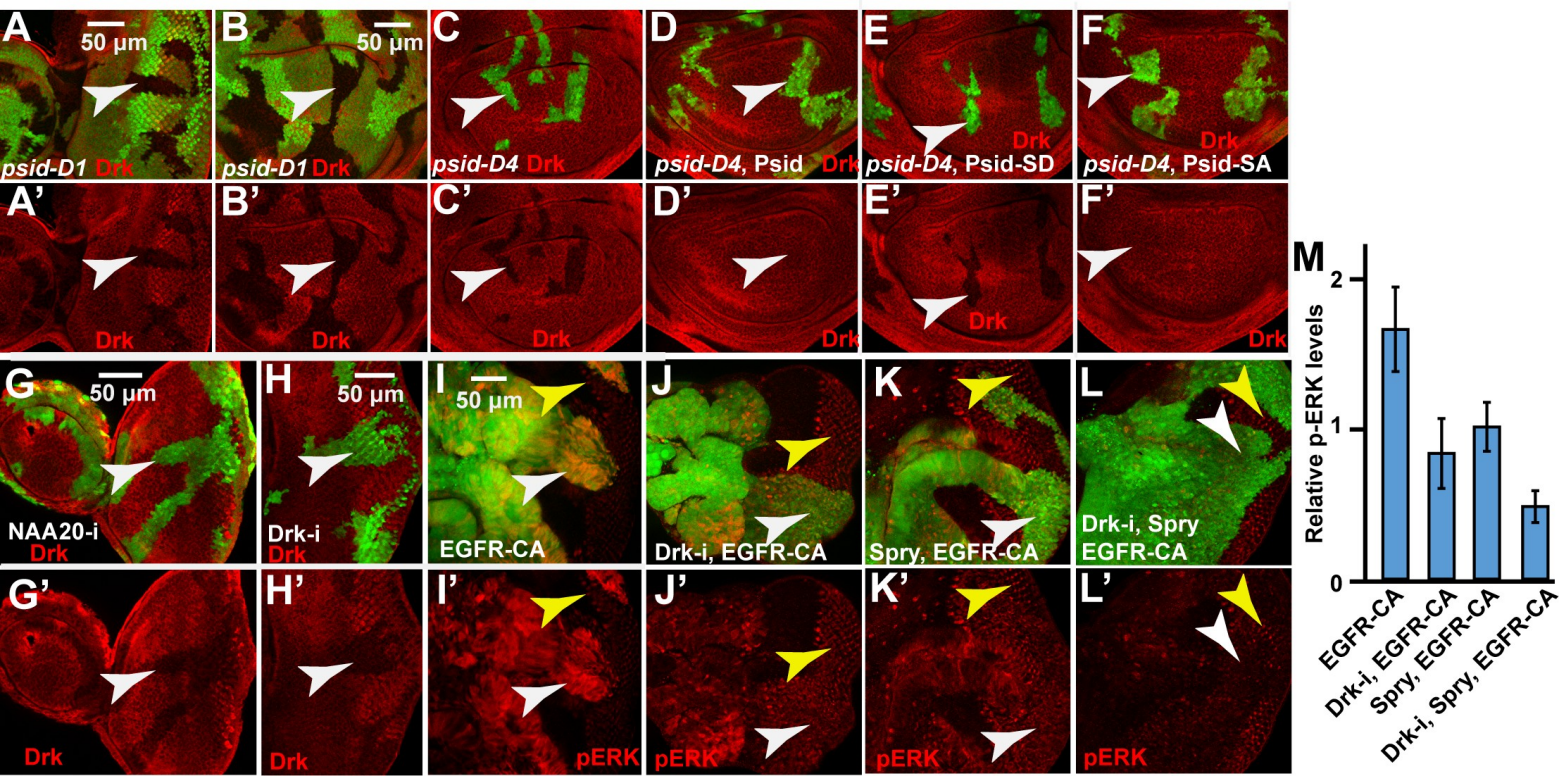
Inactivation of NatB destabilized Drk, which mediated activated EGFR-induced MAPK activation. (A-B) *psid-D1* mutant clones, marked by lack of GFP expression (pointed by arrowheads), show decreased Drk level (anti-Drk staining, red) in both eye discs (A) and wing discs (B). (C-F) MARCM clones of *psid-D4* in wing discs, marked by GFP expression (pointed by arrowheads), showed decreased Drk level (C) which was rescued by expressing WT Psid (D) or nonphosphorylatable Psid^SA^ (S678A) (F) but not the phosphomimetic mutant Psid^SD^ (S678D) (E). (G-H) NAA20 RNAi (G) or Drk RNAi (H) eye disc clones, which was generated with eyeflp-CoinGal4 and labeled with GFP (pointed by arrowheads), showed decreased Drk level. (I-M) Drk RNAi (J) reduced the level of activated EGFR-induced pERK (I), which was further inhibited by the expression of Spry (K-L). Average pERK levels normalized by the endogenous pERK in posterior eye disc and standard deviations were shown in (M) (N≥4 for each genotype). White arrowheads point to cells with activated EGFR expression and yellow arrowheads point to corresponding WT eye disc areas.

We first characterized an antibody against Drk [36]. We found Drk RNAi clones decreased Drk protein levels (Fig 4H and 4H’), indicating that the anti Drk antibody specifically recognized endogenous Drk protein. Significantly decreased Drk levels were observed in *psid* mutant clones in both eye and wing discs (Fig 4A-4B’, white arrows). Similarly, *psid* MARCM clones also significantly reduced Drk levels (Fig 4C and 4C’). Furthermore, expressing Psid^WT^ or Psid^SA^ mutant can rescue Drk levels in *psid* mutant clones while expressing the NAA20-binding defective Psid^SD^ mutant failed to rescue (Fig 4C-4F’). These results suggest that the observed effect of *psid* mutation on Drk is mediated by the Psid/NAA20 complex. Consistent with this, knockdown of NAA20 using RNAi significantly decreased Drk protein level (Fig 4G and 4G’) but not Drk mRNA level (S4A Fig). These results show that inactivation of NatB decreased levels of Drk protein posttranscriptionally.

To determine the role of Drk levels on EGFR signaling, we determined the effect of Drk knockdown on activated EGFR-induced MAPK activation. While activated EGFR induced much higher pERK levels than the endogenous pERK observed in posterior eye discs (Fig 4I-4I’ and 4M, compare white and yellow arrowheads), Drk-RNAi significantly reduced this EGFR induced pERK levels to that similar to endogenous pERK levels (Fig 4J-4J’ and 4M, compare white and yellow arrowheads). In addition, expression of Spry in conjunction with knockdown Drk further inhibited EGFR-induced MAPK activation (Fig 4I-4L’ and 4M). Therefore, reduction of Drk levels can significantly inhibit EGFR-induced MAPK activation.

### Psid regulates Drk through its N-terminal sequences

**Fig 5:**
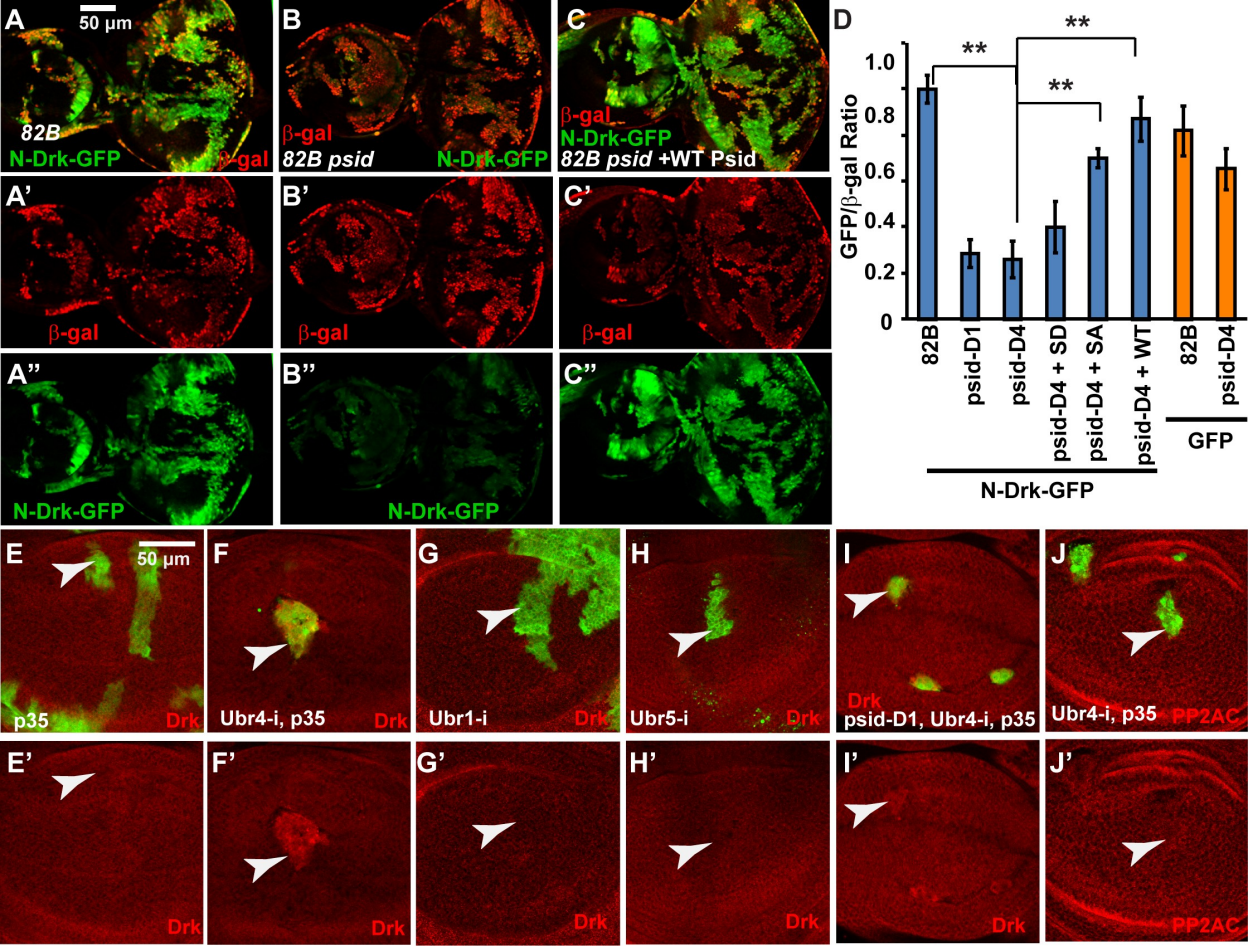
NatB-inactivation decreased Drk levels through its N-terminal sequences, which was regulated by the N-end rule E3 ubiquitin ligase POE/UBR4. (A-C) N-Drk-GFP (GFP tagged with a 10 amino acids N-terminal from Drk, green) and LacZ (β-galactosidase staining, red) were co-expressed in FRT control (A) or *psid-D4* MARCM clones (B-C) with or without co-expressed Psid rescue construct. *psid-D4* mutant clones (B) show similar levels of β-gal but decreased level of N-Drk-GFP in comparison to FRT control (A). (C) Expression of WT Psid rescued the level of N-Drk-GFP in *psid-D4* mutant clones. (D) Levels of N-Drk-GFP or WT GFP, normalized with β-gal level, in MARCM clones with indicated genotype were quantified and the average and standard deviation were shown (N≥4 for each genotype; ** indicates P<0.00001, t test). Data were collected from eye discs containing MARCM clones similar to those shown in (A-C). (E-J) Wing discs containing FRT control or *psid* MARCM clones marked by GFP expression (pointed by arrowheads). POE/Ubr4 RNAi clones were generated with the expression of p35 to prevent cell death. p35 expression alone did not influence Drk level (E). Ubr4/POE RNAi increased the Drk level in FRT control clones (F). In contrast, Ubr1 RNAi (G) or Ubr5 RNAi (H) clones did not affect Drk levels. Ubr4/POE RNAi restored the Drk level in *psid-D1* clones (I) but did not influence the level of PP2AC (J), suggesting a specific effect of Ubr4 on Drk but not PP2AC degradation.

Nt-acetylation alters the N-terminus of proteins, which can affect protein stability through the N-end rule pathway [37,38]. There are two branches of the N-end rule pathway: the Arg/N-end rule pathway and the Ac/N-end rule pathway, which target the degradation of proteins with nonacetylated or acetylated N-terminus, respectively [30,37]. To test the hypothesis that the observed effect of NatB on Drk is regulated through its N-terminal sequences, we generated a GFP protein tagged with Drk N-terminal 10 amino acids (N-Drk-GFP) and expressed it together with the β-gal control using the MARCM system. The ratio of GFP to β-gal levels were determined in WT or *psid* mutant background and compared. In comparison to the WT control, mutation of either *psid*^*D1*^ *or psid*^*D4*^ significantly decreased levels of N-Drk-GFP (Fig 5A-5B” and 5D, p<0.00001). In contrast, *psid*^*D4*^ only slightly affected the WT control GFP that do not have the N-Drk tag (Fig 5D, p = 0.01). Furthermore, the reduction in the level of GFP can be strongly rescued by expressing Psid^WT^ and Psid^SA^ but marginally by the NAA20 binding defective Psid^SD^ (Fig 5A–5D). These results, which are similar to the observed effects of Psid on endogenous Drk protein levels as shown in Fig 4C-4F’, suggest that NatB regulates Drk protein stability through its N-terminal sequences.

### Drk is degraded by the N-end rule E3 ubiquitin ligase Poe/UBR4

Nonacetylated destabilizing Nt-residues are recognized by the N-recognins of the Arg/N-end rule pathway, which induce target protein ubiquitination and degradation. N-recognins for the Arg/N-end rule pathway were found to be the UBR-box containing E3 ubiquitin ligases that are conserved from yeast to mammals [30,37,38]. The UBR box is essential for the binding of the N-terminal destabilizing residues and the recognition of the positively charged N-terminal NH3+ group is critical [39]. Interestingly, of the seven UBR box containing E3 ubiquitin ligases found in mammalian genomes, only four (Ubr1, Ubr2, Ubr4, and Ubr5) were found to be N-recognins [37], which correspond to the three UBR-box proteins (Ubr1, Ubr4, and Ubr5) in *Drosophila*.

The observed effects of NatB on Drk regulation prompted us to test whether fly Ubr1, Ubr4, or Ubr5 affects Drk levels after RNAi knockdown. As cells with Ubr4/Poe knockdown were mostly eliminated in developing discs, baculovirus p35 was expressed together with Ubr4 RNAi to inhibit cell death. Ubr4 knockdown with p35 expression significantly increased Drk levels in WT background (Fig 5F and 5F’), while p35 expression alone did not significantly affect Drk levels (Fig 5E and 5E”). Furthermore, Ubr4 knockdown with p35 expression restored Drk levels in *psid* mutant clones (Fig 5I and 5I’, compare with 4B–4C’). On the other hand, knockdown Ubr1 or Ubr5 did not affect Drk levels (Fig 5G and 5H’) and knockdown Ubr1 did not affect Drk levels in *psid* mutant background either (S4B–S4C’ Fig). These results are consistent with the idea that Drk protein degradation is regulated by the N-end rule E3 ubiquitin ligase Ubr4.

Taken together, results from our studies suggest a model that NatB regulates Drk protein stability through modification of its N-terminus, which alters its recognition by the N-end rule pathway. It should be pointed out that our results are mainly derived from genetic studies examining the effects of different genotype clones. Additional biochemical studies will be needed to confirm that Drk is directly regulated by NatB and Ubr4.

### NatB inactivation increases the levels of PP2AC, which inhibits MAPK activation in developing imaginal discs

While PP2A can potentially dephosphorylate multiple components of the Ras/MAPK pathway and exerts both negative and positive regulations, knockdown of PP2A subunits in *Drosophila* cells enhanced insulin-induced MAPK activation and reducing the gene dosage of PP2AC, the catalytic subunit of PP2A, stimulates activated Ras induced signaling in eye tissues [40,41]. These observations suggest that PP2A has an overall negative effect on RTK/Ras induced MAPK activation *in vivo*.

**Fig 6:**
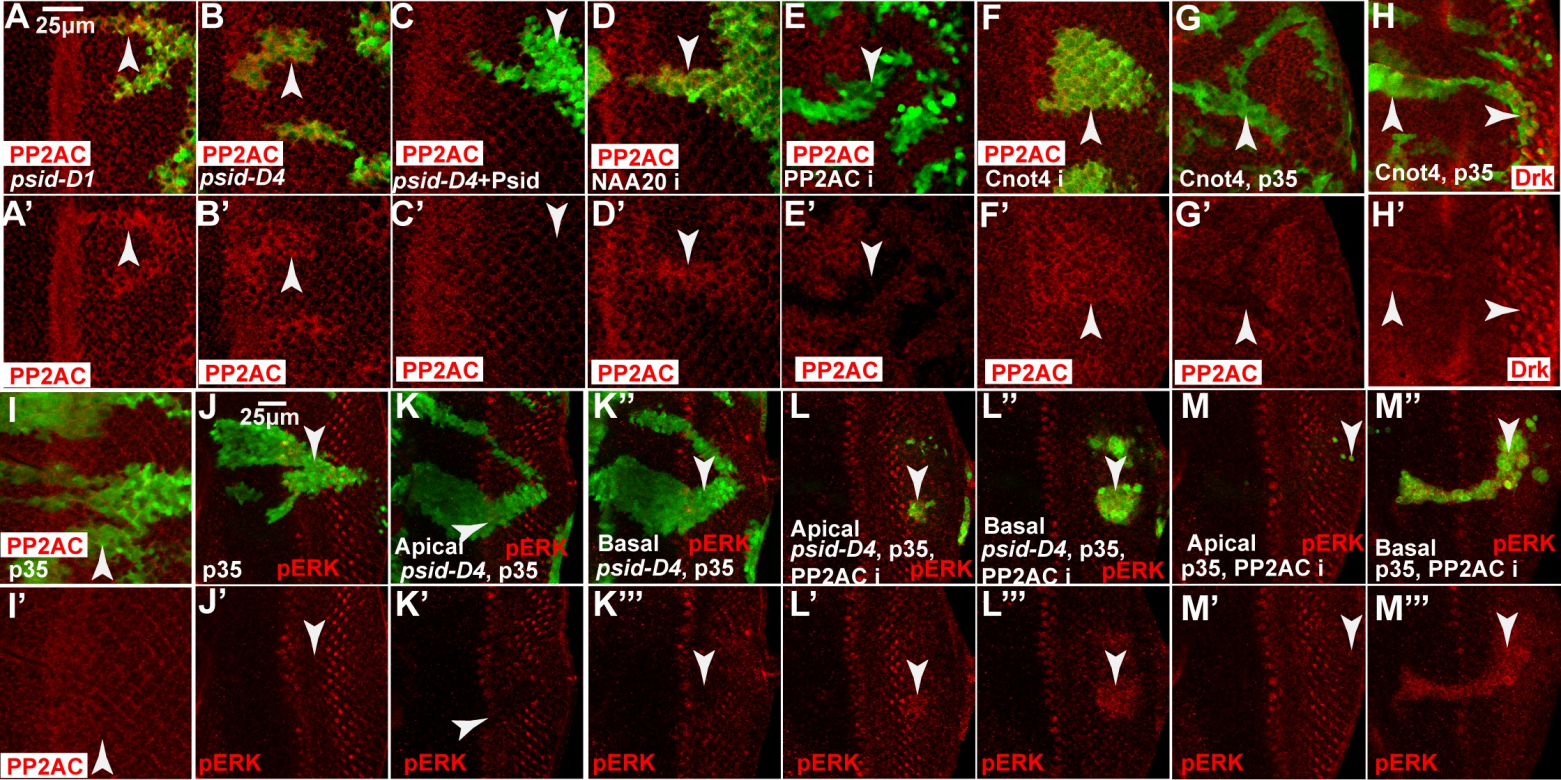
Inactivation of NatB increased levels of PP2AC, which contributes to the inhibition of MAPK activation by *psid* mutation. (A-C) Increased PP2AC level (anti-PP2AC staining, red) was observed in both *psid-D1* (A) and *psid-D4* (B) MARCM clones (marked by GFP expression, pointed by arrowheads) in eye discs. Expression of WT Psid (C) reduced PP2AC levels in *psid-D4* MARCM clones to background levels. (D) NAA20 RNAi clone generated with eyeflp-CoinGal4 and marked by GFP expression (pointed by arrowheads) showed increased PP2AC level. (E) PP2AC RNAi expression in FRT82B MARCM clones (pointed by arrowheads) decreased PP2AC levels in eye disc. (F) Cnot4 RNAi clone (pointed by arrowheads) generated with eyeflp-CoinGal4 and marked by GFP expression showed increased PP2AC level. (G-I) GFP and p35 were expressed together with Cnot4 to prevent cell death and to mark the expressing cells (pointed by arrowheads). Cnot4 overexpression decreased PP2AC protein level (G) but did not significantly affect Drk level (H), while expressing p35 alone did not affect PP2AC level (I). (J-M) Apical or Basal sections of representative eye discs containing *psid* or FRT control MARCM clones marked by GFP expression (pointed by arrowheads). p35, which did not affect pERK (J), was used to prevent the elimination of PP2AC RNAi clones. *psid-D4* clones showed decreased pERK level (K). PP2AC RNAi expression cells in *psid-D4* clones (L-L”‘) or in FRT control clones (M-M”‘) were mostly located at the basal section (L”-L”‘ and M”-M”‘) and showed increased pERK levels.

We first used PP2AC RNAi to identify an antibody that can recognize the endogenous fly protein. As shown in Fig 6E, PP2AC RNAi significantly decreased PP2AC protein signal, indicating that this PP2AC antibody can specifically detect the endogenous protein. Interestingly, MARCM clones with either *psid*^*D1*^ or *psid*^*D4*^ mutation showed increased PP2AC protein levels (Fig 6A–6B’). The increased PP2AC protein was dependent on the *psid* mutation since expression of WT Psid blocked the increased PP2AC levels (Fig 6C and 6C’). Furthermore, knockdown of NAA20 significantly increased PP2AC protein level (Fig 6D and 6D’) but not PP2AC mRNA level (S5C Fig). These results showed that inactivation of NatB activity increased PP2AC protein level posttranscriptionally.

Overexpression of PP2AC in mouse heart was shown to increase PP2AC level, reduce the phosphorylation level of its targets, and impair cardiac function [42]. To determine whether increased PP2AC levels contribute to reduced MAPK activation in *psid* mutant clones, we tested effects of knockdown PP2AC in *psid* mutant or control MARCM clones. Since PP2AC knockdown with *psid* mutation induced significantly levels of cell death, baculovirus p35 protein, which does not affect pERK levels when expressed alone (Fig 6J), was expressed in conjunction with PP2AC knockdown in these experiments. While reduced pERK levels observed in *psid* mutant clones (Fig 6K and 6K”‘), *psid* mutant cells with PP2AC knockdown showed increased pERK levels, particularly in the basal region of the eye disc where these cells were mostly observed (Fig 6L and 6L”‘). In addition, PP2AC-RNAi cells also showed similarly increased pERK levels (Fig 6M and 6M”‘). These results, which are consistent with the previous finding that PP2AC has an overall negative effect on RTK/Ras induced MAPK activation [40], suggested that increased PP2AC contributes to the inhibition of MAPK activation by *psid* mutation.

### PP2AC and Drk are regulated by distinct branches of the N-end rule pathways

The increased level of PP2AC observed in NatB inactivated cells led us to hypothesize that the N-terminally acetylated PP2AC but not the N-terminally unmodified PP2AC is preferentially degraded by the Ac/N-end rule pathway. Two Ac/N-recognins Doa10 and Not4 have been identified in yeast and the Doa10 homolog Teb4 has been shown to function as a mammalian Ac/N-recognins [30,43], suggesting that the Ac/N-end rule pathway is also conserved. The Doa10/Teb4 and Not4 homologs in *Drosophila* are CG1317 and Cnot4, respectively. We generated an allele of fly Teb4 that deleted a significant portion of the open reading frame including the initiation ATG (S5A Fig). Clones of cells with *teb4/CG1317* mutation were quite small but did not affect PP2AC levels (S5B and S5B’ Fig). These results suggest that Teb4/CG1317 does not significantly affect PP2AC degradation. On the other hand, inactivating the fly Not4 E3 ubiquitin ligases homolog Cnot4 with RNAi significantly increased levels of PP2AC protein (Fig 6F and 6F’) but not PP2AC mRNA (S5C Fig). Additionally, overexpression of Cnot4 significantly decreased the basal levels of PP2AC (Fig 6G and 6G’). These results suggest that Cnot4 but not Teb4/CG1317 contribute to the degradation of PP2AC.

The above results suggested that PP2AC is degraded by the Ac/N-end rule E3 ubiquitin ligase Cnot4 while Drk is degraded by the Arg/N-end rule pathway E3 ubiquitin ligase Ubr4. We further determined whether PP2AC could also be significantly affected by the Arg/N-end rule pathway and whether Drk significantly affected by the Ac/N-end pathway. Ubr4 knockdown did not significantly affect PP2AC levels (Fig 5J and 5J’) despite significantly increased the levels of Drk (Fig 5F and 5F’). In addition, knockdown of Ubr1 and Ubr5, the other two fly N-recognins of the Arg/N-end rule pathway, did not significantly affect PP2AC levels either (S5D–S5E’ Fig). These results suggest that PP2AC is not significantly affected by the Arg/N-end rule pathway E3 ubiquitin ligases. On the other hand, overexpression of Cnot4 did not significantly affect Drk levels despite significantly reduced PP2AC levels (Fig 6G and 6H’). In addition, mutation of Teb4/CG1317 did not significantly affect Drk levels either (S5F Fig). These results show that Drk protein level was not significantly affected by the Ac/N-end rule E3 ubiquitin ligases.

Taken together, our results are consistent with the model that PP2AC is regulated by the Ac/N-end rule pathway E3 ubiquitin ligase Cnot4 while Drk is preferentially targeted for degradation by the Arg/N-end rule pathway E3 ubiquitin ligase Ubr4.

### NatB inactivation decreased the level of MAPK, another highly conserved positive component of the EGFR/MAPK pathway

The above results showed that NatB inactivation inhibited EGFR/MAPK pathway by decreasing the levels of the conserved positive regulator Drk while increasing the levels of the conserved negative regulator PP2AC. To gain better understanding of how NatB/N-end rule pathway modulate EGFR/MAPK signaling, we further characterized the effect of NatB on other potential targets with available antibodies, MAPK, Spry, and PTP-ER.

MAPK has high level of overall sequence conservation except the N-terminal region. Interestingly, a previous study showed that MAPK is degraded by the Poe/Ubr4 E3 ubiquitin ligase, suggesting that MAPK is regulated by the Arg/N end rule pathway [44]. Therefore, it is very interesting to determine whether NatB affects MAPK levels.

**Fig 7:**
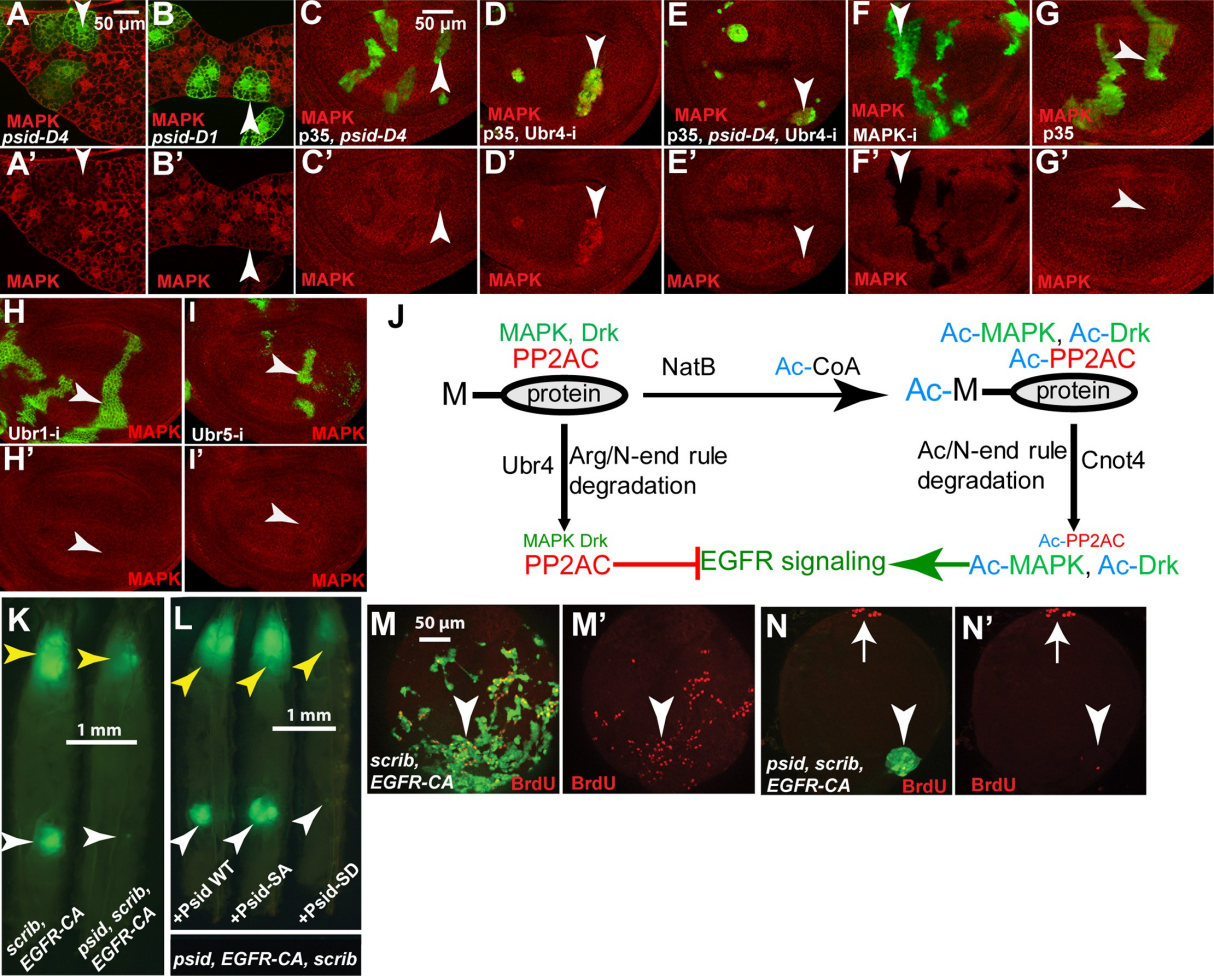
NatB inactivation decreased the level of MAPK and proposed model of EGFR/MAPK signaling regulation by NatB and the N-end rule pathways. (A-B) *psid-D1* (A) or *psid-D4* (B) clones in 3rd instar fat body, which were marked by GFP expression (pointed by arrowheads), showed decreased MAPK levels (anti-MAPK staining, red). (C) *psid-D4* clones in wing disc, marked by GFP expression (pointed by arrowheads), showed decreased MAPK level. (D-E) Ubr4 RNAi clones, marked by GFP expression (pointed by arrowheads), showed increased MAPK level (D). Ubr4 RNAi also restored the level of MAPK in *psid-D4* clones (C, E). p35 expression, which did not affect MAPK levels (G), was used to block the death of Ubr4 RNAi cells. (F) The specificity of the anti-MAPK antibody. Reduced MAPK staining was observed in MAPK RNAi clones marked by GFP expression (pointed by arrowheads). (H-I) Ubr1 RNAi (H) or Ubr5 RNAi (I) clones (pointed by arrowheads) did not significantly affect MAPK levels. (J) A proposed model of EGFR/MAPK signaling regulation by NatB and the N-end rule pathways through inversely modulating the levels of positive and negative pathway components is shown. Drk/Grb2 and MAPK, two positive components of the EGFR signaling pathway are targeted for degradation by the Arg/N-end rule pathway and E3 ubiquitin ligase Ubr4 while PP2AC, a negative regulator of EGFR signaling pathway, is targeted for degradation by the Ac/N-end rule pathway E3 ubiquitin ligase Cnot4. The levels of acetylation of these proteins are controlled by NatB activity and the key rate-limiting substrate Ac-CoA. High NatB activity and high Ac-CoA level will increase the levels of Nt-acetylated Grb2/Drk, MAPK, and PP2AC, leading to high EGFR signaling due to elevated levels of Grb2/Drk and MAPK (positive components of the pathway) and reduced levels of PP2AC (negative component of the pathway). In contrast, low NatB activity and low Ac-CoA levels will reduce the levels of Nt-acetylated Grb2/Drk, MAPK, and PP2AC, leading to reduced EGFR signaling due to decreased levels of Grb2/Drk and MAPK (positive components of the pathway) and increased levels of PP2AC (negative component of the pathway). (K) *psid* mutation inhibited Ey-FLP induced MARCM clones of activated EGFR expression (labelled with GFP) in *scrib* mutant clones. Yellow and white arrows point to tumor growth in the head region and the male gonads, respectively. (L) Expression of WT Psid or the Psid-SA mutant but not the NAA20-binding defective Psid-SD mutant restored tumor growth, suggesting the NatB activity is required for activated EGFR-induced tumor growth. (M-N’) *psid* mutation significantly inhibited activated EGFR-induced cell proliferation in male gonads. *scrib* mutant clones with activated EGFR expression (pointed by arrowhead) were highly proliferative with high level of BrdU incorporation (M-M’, red). In contrast, mutation of *psid* significantly inhibited BrdU incorporation in *EGFR scrib* cells (N-N’, arrowhead pointed GFP positive cells). Arrows in N-N’ point to the normal proliferating cells in the anterior pole.

We found that inactivation of NatB with *psid* mutation moderately reduced MAPK levels in wing disc (Fig 7C), using an anti-MAPK antibody that can detect the endogenous MAPK protein (Fig 7F). In addition, significant reduced MAPK protein levels were also observed in both *psid*^*D1*^ or *psid*^*D4*^ mutant clones in fat body (Fig 7A–7B’). Therefore, NatB also regulates MAPK levels. In support of the previous report that MAPK is degraded by Ubr4, knockdown of Ubr4 with p35 expression significantly increased MAPK levels (Fig 7D and 7D’), while p35 expression alone or knockdown of Ubr1 or Ubr5 had no effect (Fig 7G–7I’). In addition, knockdown of Ubr4 also blocked *psid* mutation induced-decrease in MAPK levels (Fig 7C–7E’). Taken together, NatB activity also increased the level of MAPK, another conserved positive component of the EGFR signaling pathway.

### NatB inactivation did not affect the posttranscriptional level of Spry, a component of the EGFR/MAPK pathway that is not highly conserved

In contrast to the highly conserved sequences of Drk and PP2AC, Spry has low overall sequence conservation except in a cysteine-rich region near the C-terminus [45]. Staining with an antibody against Spry [45] detected elevated Spry levels in posterior eye discs that was significantly reduced by Spry RNAi (S6A and S6A’ Fig, white arrowheads). In contrast, the background signal in anterior eye disc regions was not affected by Spry-RNAi (S6A and S6A’ Fig, yellow arrowheads). These observations consistent with reports that Spry expression is dependent on RTK signaling, which is activated in the posterior region of developing eye [45,46]. Decreased Spry levels were detected in *psid* mutant clones (S6B Fig). Since *psid* mutation reduces EGFR/MAPK signaling, which in turn regulates Spry expression, the observed decreased Spry level in posterior eye disc could be due to reduced Spry expression in *psid* clones. To further characterize whether *psid* mutation affects Spry level independent of transcription, we co-expressed Spry-HA with GFP in *psid* or control MARCM clones. As shown in S6 Fig, similar levels of Spry-HA normalized by GFP signal were observed in the *psid* and the control MARCM clones (S6C–S6E Fig). These results showed that *psid* mutation did not significantly affect the levels of Spry posttranscriptionally, a negative regulator of RTK signaling that does not have high sequence conservation.

Of the other two remaining potential NatB targets that function between EGFR and MAPK, PTP-ER and Sur-8, only PTP-ER has available antibodies [47]. However, although the PTP-ER antibodies can recognize PTP-ER protein on western blots [47], staining imaginal discs with either monoclonal antibody showed only background signal, which was not affected by PTP-ER RNAi constructs (S6F–S6G’ Fig). Therefore, we could not use these antibodies to determine whether *psid* mutation affects PTP-ER levels.

### *psid* mutation inhibited activated EGFR-induced tumor growth

Mutation, increased expression, and amplification of EGFR that deregulate the EGFR signaling are linked with the development of a variety of human cancers [48]. Therefore, it is of significant interest to determine whether *psid* inactivation can inhibit EGFR signaling-induced tumor growth. In *Drosophila*, expression of activated EGFR or activated Ras in conjunction with *scrib* mutation induces cephalic and male gonadal tumors when Ey-FLP was used to induce MARCM clones (Fig 7K) [49,50,51]. Interestingly, *psid* mutation significantly inhibited activated EGFR-induced tumor growth in both regions (Fig 7K, Yellow and white arrowheads, tumor cells were marked by GFP). The gonadal tumor was initiated by Ey-FLP induced *scrib* MARCM clone in the ‘terminal body’ (TB) cells at the posterior pole, which grows and spreads to the anterior [51]. The *EGFR*^*CA*^ *scrib* mutant cells were highly proliferative as shown by the large numbers of BrdU incorporating cells (Fig 7M and 7M’). Inactivation of *psid* significantly inhibited the proliferation of *EGFR*^*CA*^ *scrib* mutant cells (Fig 7N and 7N’, GFP positive cells pointed by arrowhead) despite normal cell proliferation in the anterior pole can be easily detected (Fig 7N and 7N’, GFP negative cells pointed by arrow). Furthermore, expressing Psid^WT^ or Psid^SA^ but not the NAA20 binding defective Psid^SD^ mutant restored *EGFR*^*CA*^ *scrib* tumor growth in *psid* mutant background (Fig 7L). These results suggest that Psid-regulated NatB activity is required for activated EGFR-induce tumor growth.

## Discussion

Our results suggest a model by which NatB activity modulates EGFR/MAPK signaling by regulating the levels of multiple highly conserved pathway components posttranscriptionally (Fig 7J). The N-terminus of protein have been shown to influence protein stability through the N-end rule pathway with branches that selectively degrade N-terminally acetylated protein or N-terminally unmodified protein [30]. Interestingly, we found that NatB targets that are highly conserved positive components of EGFR signaling pathway are selectively targeted for degradation by the Arg/N end rule E3 ubiquitin ligase Ubr4 (Fig 7J). In contrast, NatB target that is a highly conserved negative component of the pathway, PP2AC, is selectively targeted for degradation by the Ac/N end rule E3 ubiquitin ligase Cnot4 (Fig 7J). As E3 ubiquitin ligases for the two branches of the N-end rule pathway selectively recognize the positively charged non-acetylated N-terminus or the acetylated N-terminus that lacks the positive charge, respectively, NatB activity, which shifts the level of protein Nt-acetylation, will potentially lead to coordinated changes in the levels of positive and negative components of the pathway (Fig 7J, S7A and S7B Fig). In this model, inhibition of NatB activity would result in the loss of N-terminal acetylated Drk/Grb2 and MAPK and the degradation of the N-terminal non-acetylated proteins, leading to decreased levels of positive components (S7A Fig). Furthermore, inhibition of NatB activity would also result in the accumulation of N-terminal non-acetylated PP2AC, which would prevent its degradation by the Ac/N end rule E3 ubiquitin ligase Cnot4 and result in the increased levels of this negative pathway component (S7A Fig). It is likely that the inverse changes in the levels of multiple positive and negative components of the pathway underlie NatB activity-mediated regulation of EGFR signaling (Fig 7J, S7A and S7B Fig). In addition, since many receptor tyrosine kinase (RTK) signaling share these same components, it is possible that these RTK signaling can be similarly affected by N-terminal acetylation and N-end rule pathway.

Because Nt-acetylation is believed to be largely co-translational and without a corresponding deacetylase, Nt-acetylation is often regarded as a constitutive, irreversible, and static modification that is not suited to serve regulatory functions. This study, however, suggest N-terminal acetylation by NatB may provide important regulatory function. First, NatB activity mainly regulates the level of highly conserved pathway components. Second, NatB activity coordinately regulates the levels of positive and negative components of the EGFR signaling pathway. Third, as knockdown of Ubr4 significantly elevated levels of both Drk/Grb2 and MAPK and as Ubr box proteins specifically recognizes N-terminal non-acetylated proteins, these results suggest that significant levels of endogenous Drk/Grb2 and MAPK are without N-terminal acetylation in normal growth conditions and are targeted for degradation by Ubr4. It is likely that these proteins are partially Nt-acetylation under normal growth conditions, which could potentially be regulated by increasing or decreasing the level of NatB activity or Acetyl-CoA, a key substrate of N-terminal acetylation.

Acetyl-CoA has been suggested as a central metabolite and second messenger of the cellular energetic state [52]. The levels of Acetyl-CoA, which is influenced by the availability of nutrients and growth factors, can vary significantly in cells and influence the levels of protein acetylation by various acetyl transferases [52,53]. Indeed, Acetyl-CoA level was shown to regulate the N-terminal acetylation of several NatA targets, which modulates the sensitivity of cells to apoptosis [54]. As the Km for NatB from *Candida* is quite high (around 50 μM) [55], it is likely that N-terminal acetylation by NatB will also be influenced by Acetyl-CoA levels, which will potentially link the regulation of EGFR signaling with cellular energetic status.

In addition to Acetyl-CoA level, NatB activity may also be regulated. It was shown that a conserved serine in Psid just downstream of the NAA20 interaction domain regulated NatB complex formation in a phosphorylation-dependent manner (Stephan et al., 2012). Similarly, we found that expressing the phosphorylation resistant but not the phosphorylation mimic form of Psid could rescue the effect of *psid* mutation on EGFR signaling. These results suggest NatB activity is regulated by cellular signaling pathways through phosphorylation. Taken together, our study suggest that the level of N-terminal acetylation is potentially regulated by cellular signaling and nutrient status, which would in turn regulate the strength of EGFR signaling output by coordinately regulated the levels of multiple positive and negative regulators of the pathway (S7A and S7B Fig). Further studies will be needed to test these possibilities.

The high sequence conservation of the EGFR signaling components, such as Grb2 and PP2AC, which are regulated by NatB and N-end rule pathways suggest that the mechanisms we uncovered in *Drosophila* is likely conserved in mammalian systems. Indeed, a recent study found that inhibition of NatB significantly decreased EGF-induced ERK activation in human liver cancer cells [56]. As mutation or overexpression of EGFR or Ras that deregulate EGFR/MAPK signaling are quite common in human cancers and NatB subunits are significant unfavorable prognostic markers for human cancers [57,58,59], our results could potentially provide a new strategy to develop therapeutic interventions for these cancers.

The regulation of MAPK activity by NatB described in this study also provides a mechanism by which Nt-acetylation by NatB is required to ensure olfactory receptor neuron (ORN) survival as described previously [29]. It was shown that the phospho-resistant Psid^SA^ mutant rescued the *psid* cell-loss phenotype while the phosphomimetic Psid^SD^ mutant failed to rescue [29]. In this study, we showed that the phospho-resistant Psid^SA^ but not the phosphomimetic Psid^SD^ was able to rescue *psid* mutation-mediated MAPK inhibition and *rbf psid* synergistic cell death.

We showed that *psid* mutation significantly inhibited pERK level induced by activated EGFR and Ras but not activated Raf. At first glance, these results do not seem to be consistent with the result that MAPK is also regulated by NatB. One possible explanation is that the unphosphorylated MAPK level was not limiting and therefore the moderate change in MAPK level did not significantly affect Raf-induced MAPK activation. Another possible explanation is that the effect of reduced MAPK levels was compensated by the effect of increased levels of PP2AC. It was shown that activation of Raf by Ras-GTP involve activating phosphorylation as well as inhibitory phosphorylation by ERK feedback regulation [60]. It is possible that in experiments involving activated Raf-induced MAPK activation, the main function of PP2A was to remove the inhibitory phosphorylation on Raf. In contrast, in experiments involving activated Ras-induced MAPK activation, the dominant function of PP2A was to remove the activating phosphorylation. Consistent with this, reducing the PP2AC gene dosage was found to impair signaling from activated Raf but stimulate signaling from activated Ras [40]. Therefore, *psid* mutation induced increased PP2AC potentially compensated decreased MAPK levels, resulting no obvious inhibition of activated Raf-induced dpERK level.

## Materials and methods

### Drosophila stocks and genetics

The following fly stocks were used in this study: *rbf*^*15aΔ*^ [19]; *psd-D1*(BL41122, BL indicating Bloomington Drosophila stock center); *psd-D4* (BL41123), UAS-Rbf RNAi (BL36744), *scrib* ^673^ (BL41175), UAS-NAA20 RNAi (BL36899), UAS-Drk RNAi (BL41692), UAS-Poe RNAi (BL32945), UAS-PP2AC RNAi (BL 27723), UAS-Cnot4 RNAi (BL42513), UAS-Cnot4 (BL22246), UAS-p35 (BL5072, BL5073), UAS-MAPK RNAi (BL34855), UAS-Ubr5 RNAi (BL32352), UAS-Ubr1 RNAi (BL31374), UAS Psid-WT [28], UAS-Psid-S678D and UAS-Psid-S678A [29], aos-lacz and UAS-EGFR^*CA*^ [24], UAS-ras^*v12*^ (BL64196), UAS-raf^*gof*^ (BL2033), UAS-GFP (BL5431), CoinFLP-Gal4-UAS-GFP (BL58751), UAS-Dcr2 (BL58757), UAS-sprouty RNAi (BL36709), UAS-PTP-ER RNAi (BL53311), and UAS-sprouty [46].

Main genetic technologies used in this study include: FLP/FRT system to generate regular loss of function mosaic clones [61]; MARCM system to generate mosaic clones with both mutation and ectopic expression [62]; UAS/Gal4 and Flp-out or CoinFLP system to induce ectopic expression of RNAi in clones or in whole eyes [63,64,65].

### Genetic screen for *rbf* synthetic lethal mutations and generation of N-Drk-GFP transgenic fly and CG1317/Teb4 deletion allele

Ethyl methanesulfonate (EMS)-induced mutant screen was carried out as described [19]. Isogenized w; *P{ry+*, *neoFRT82B}* males were used for mutagenesis, *rbf*^*15aΔ*^,*w*, *eyFLP*; *P{ry+*, *neoFRT82B} P{w+*, *Ubi-GFP} P{w+*, *Rbf-G3}* and *w*, *eyFLP*; *P{ry+*,*neoFRT82B} P{w+*, *Ubi-GFP}* stocks were used for screening and *rbf* dependence test.

The N-Drk-GFP transgenic fly, which contains the N-terminal 10 amino acid sequence from Drk (ATG GAA GCG ATT GCC AAA CAC GAT TTC TCT) fused to the N-terminus of GFP from PX458 plasmid was generated by PCR and verified by sequencing. The N-Drk-GFP fusion was cloned into the pUAST plasmid and transgenic flies were established.

The CG1317/Teb4 deletion allele was generated by crossing the P-element insertion line (BL20646) with the transposase line Δ2–3. Independent excision lines that have lost eye color were established. Deletions were identified by PCR using P element primers and primers flanking the P element. The breakpoints were determined by sequencing of the PCR products.

### Immunostaining, BrdU incorporation, and antibodies

Immunostaining was performed as previously described [24]. For dpErk staining, dissected discs were fixed with 8% formaldehyde in PBS for 1 hr. For PP2AC staining, dissected discs were fixed with 4% formaldehyde in 100mM Lysine for 1 hr on ice, and saponin was added in the blocking solution to a final concentration of 0.2% during the process of blocking and primary antibody incubation. For Brdu incorporation of male gonads, male gonads were dissected from larvae with growing tumors at 7–8 day after egg laying in Schneider’s medium. Samples were incubated with BrdU (75 μg/ml in Schneider’s medium) at RT for 1 hr, washed with PBS, and fixed with 4% formaldehyde in PBS for 30 minutes at RT, followed by postfixing with 4% formaldehyde in PBS with 0 .6% Tween 20 for 30 minutes at RT. These samples were washed with DNase I buffer, followed by incubation with DNase I (100 U/500 μl) for 1 hr and wash with PBST (0.3% triton X-100). Primary antibodies used in this study: rabbit anti-cleaved Caspase-3 (C3, 1:400 from Cell Signaling, Cat #9661), rabbit anti dpErk (1:400, Cell signaling, Cat #4370), rabbit anti-MAPK (1:500, Cell signaling, Cat #4695), mouse anti-PP2AC (1:400, Santa Cruz Biotechnology, sc-80665), rabbit anti-Drk (1:1000) [36], Guinea pig anti-senseless (1:1000, gift from Dr. Hugo Bellen), rat anti-Elav (1:100, DSHB, 7E8A10), mouse anti-BrdU (1:50, DSHB, G3G4), mouse anti-β-Galactosidase (1:100, DSHB, 40-1a). Secondary antibodies are from Jackson ImmunoResearch (1:400). Samples were mounted in 70% Glycerol with 1,4-diazabicyclo[2.2.2]octane (DABCO) at 12.5 mg/mL. Samples were imaged with an AxioCam CCD camera mounted on a Zeiss Axio Imager with ApoTome using the Zeiss Axiovision software.

### Quantification of cell death levels and N-Drk-GFP levels in developing imaginal discs

Cell death level was determined by the percentage of clone area (pixels) that have above background levels of caspase 3 (C3) signal using the Histogram function in Photoshop as described previously [20]. Background level of C3 signal was determined from the adjacent WT tissues that have no apoptosis. The average and standard deviation of percent cell death for each genotype discs was then determined from at least six imaginal discs and then compared. Two-way student T test was used to determine the significance of statistical differences between different genotypes.

To investigate the effect of *psidin* mutation on N-Drk-GFP levels, MARCM clones were generated and marked by LacZ expression. LacZ expression levels were determined by anti-β-gal staining and used as an internal control. Exposure time of imaging was optimized with the brightest samples and used for all samples. GFP and β-gal signal brightness was calculated using the Histogram function in Photoshop. Normalized GFP levels were showed as relative ratios of signal brightness of GFP to that of β-gal with the normalized GFP level in WT control set as 1. The average and standard deviation of relative GFP levels for each genotype discs was then determined using at least six imaginal discs and compared. Two-way student T test was used to determine the significance of statistical differences between different genotypes.

### RNA isolation and quantitative real-time PCR

Total RNA was extracted using TRI reagent (Invitrogen) from about 30 eye/antenna discs dissected from 3rd instar larvae cultured at 25°C with expression of RNAi constructs driven by eyFLP, Act>CD2>Gal4. cDNA synthesis and qRT-PCR reactions were performed as previously described [66]. Ribosomal protein gene rp49 was used as an internal control in qPCR analysis. The averages and standard deviations of at least three independent replicates were shown. Primers used were as follows: Drk for 5’-GGAAGCGATTGCCAAACAC-3’; Drk rev 5’-GCGCGATACCAATTTGAATCG-3’; PP2AC for 5’-GCAATCAGTTGACAGAGACACA-3’; PP2AC Rev 5’-CACCGGGCATTTTACCTCCT-3’; Rp49 F 5’-ACAGGCCCAAGATCGTGAAGA-3'; Rp49 R 5’- CGCACTCTGTTGTCGATACCCT-3’.

### Genotype of flies used in this study

Fig 1

w, eyFLP/Y; FRT82B,*Rps3*^*-*^, Ubi-GFP/FRT82B (panel A)

*rbf*^*15aΔ*^,w, eyFLP/Y; FRT82B, RBF-G3, *Rps3*^*-*^, Ubi-GFP/ FRT82B (panel B)

w, eyFLP/Y; FRT82B,*Rps3*^*-*^, Ubi-GFP/FRT82B, *psid* (*AE*, *or D1*) (panels C, E)

*rbf*^*15aΔ*^,w, eyFLP/Y; FRT82B, RBF-G3, *Rps3*^*-*^, Ubi-GFP/ FRT82B, *psid* (A*E*, *or D1*) (panels D, F)

eyFLP, Act>CD2>Gal4/Y; UAS-Rbf RNAi /+ or UAS-NAA20 RNAi (panels H, J)

eyFLP, Act>CD2>Gal4/Y; + or UAS-NAA20 RNAi (panels G, I)

w,eyFLP (or HsFLP)/Y; Act > y >Gal4, UAS-GFP; FRT82B, tub-Gal80/ FRT82B, *psid D4* (panels L, P)

*rbf*^*15aΔ*^, w,eyFLP (or HsFLP)/Y; Act > y >Gal4, UAS-GFP; FRT82B, RBF-G3, tub-Gal80/ FRT82B or (FRT82B, *psidin D4*) (panels K, M, O, Q)

*rbf*^*15aΔ*^,w, eyFLP (or HsFLP)/Y; Act > y >Gal4, UAS-GFP/ UAS-psidin WT; FRT82B, RBF-G3, tub-Gal80/ FRT82B, *psid D4* (panels N, R)

Fig 2

w, eyFLP /Y; FRT82B, Ubi-GFP / aos-lacz, FRT82B, *psdin psid D4* (panel A)

HsFLP; Act > y >Gal4, UAS-GFP/ + or UAS-psidin (WT, SD, or SA); FRT82B, tub-Gal80/ FRT82B,*psid D4* (panels B-E)

eyFLP, UAS-Dcr2 / +; CoinFLP-Gal4-UAS-GFP; aos-lacz, UAS-NAA20 RNAi (panel F)

*rbf*^*15aΔ*^,w, HsFLP/Y; Act > y >Gal4, UAS-GFP/ + or UAS- (EGFR^CA^, Ras^v12^, or Raf^gof^); FRT82B, RBF-G3, tub-Gal80/ FRT82B, *psid D4* (panels G-J)

Fig 3

HsFLP; Act > y >Gal4, UAS-GFP/ UAS- (EGFR^CA^, Ras^v12^, or Raf^gof^); FRT82B, tub-Gal80/ FRT82B or (FRT82B, *psid D4*) (Panels A-F)

HsFLP; Act > y >Gal4, UAS-GFP/ UAS-EGFR^CA^, UAS-psid (WT, SD, or SA); FRT82B, tub-Gal80/ FRT82B, *psid D4* (panels G-I)

eyFLP, UAS-Dcr2 / +; CoinFLP-Gal4-UAS-GFP/ EGFR^CA^; + or UAS-NAA20 RNAi (panels J-K)

Fig 4

w, HsFLP; FRT82B, Ubi-GFP / FRT82B, *psdin D1* (panels A-B)

HsFLP; Act > y >Gal4, UAS-GFP/ + or UAS-psid (WT, SD, or SA); FRT82B, tub-Gal80/ FRT82B, *psid D4* (panels C-F)

eyFLP, UAS-Dcr2 / +; CoinFLP-Gal4-UAS-GFP; UAS-NAA20 RNAi (panel G)

eyFLP, UAS-Dcr2 / +; CoinFLP-Gal4-UAS-GFP; UAS-Drk RNAi (panel H)

eyFLP, UAS-Dcr2 / +; CoinFLP-Gal4-UAS-GFP/ UAS-EGFR^CA^; + or UAS-Drk RNAi (panels I-J)

eyFLP, UAS-Dcr2 / +; CoinFLP-Gal4-UAS-GFP/ UAS-EGFR^CA^,UAS-sprouty; + or UAS-Drk RNAi (panels K-L)

Fig 5

eyFLP; Act > y >Gal4, UAS-Lacz; FRT82B, tub-Gal80/ UAS-N-Drk-GFP, FRT 82B (panel A)

eyFLP; Act > y >Gal4, UAS-Lacz / + or UAS-psid (WT, SD, or SA); FRT82B, tub-Gal80/ UAS-N-Drk-GFP, FRT 82B, *psid D4* (*or psid D1*) (panels B-D)

eyFLP; Act > y >Gal4, UAS-Lacz / Act > y >Gal4, UAS-GFP; FRT82B, tub-Gal80/ FRT 82B or (FRT82B, *psid D4*) (panel D)

HsFLP; Act > y >Gal4, UAS-GFP/UAS-p35; FRT82B, tub-Gal80/ FRT82B (panel E)

HsFLP; Act > y >Gal4, UAS-GFP/UAS-p35; UAS-Ubr4 RNAi,FRT82B, tub-Gal80/ FRT82B or (FRT82B, *psid D1*) (panels F, I, J)

HsFLP; tub-Gal80, FRT40A/ FRT40A; Act > y >Gal4, UAS-GFP / UAS-(Ubr1 RNAi or Ubr5 RNAi) panels (G-H)

Fig 6

HsFLP; Act > y >Gal4, UAS-GFP; FRT82B, tub-Gal80/ FRT82B, *psid D4 or* (*psid D1*) (panels A-B)

HsFLP; Act > y >Gal4, UAS-GFP/ UAS-psid WT; FRT82B, tub-Gal80/ FRT82B, *psid D4* (panel C)

eyFLP, UAS-Dcr2 / +; CoinFLP-Gal4-UAS-GFP; UAS-NAA20 RNAi (panel D)

w,eyFLP; Act > y >Gal4, UAS-GFP; FRT82B, tub-Gal80/UAS-PP2AC RNAi, FRT82B (panel E)

eyFLP, UAS-Dcr2 / +; CoinFLP-Gal4-UAS-GFP/ UAS-Cnot4 RNAi (panel F)

HsFLP; Act > y >Gal4, UAS-GFP, UAS-p35/ + or UAS-Cnot4; FRT82B, tub-Gal80/ FRT82B (panels G-I)

HsFLP; Act > y >Gal4, UAS-GFP/ UAS-p35; FRT82B, tub-Gal80/ FRT82B or [(FRT82B, *psid D4*), (UAS-PP2AC RNAi, FRT82B, *psid D4*) or (UAS-PP2AC RNAi, FRT82B)] (panels J-M)

Fig 7

HsFLP; Act > y >Gal4, UAS-GFP; FRT82B, tub-Gal80/ FRT82B, *psid D4 or* (*psid D1*) (panels A-B)

HsFLP; Act > y >Gal4, UAS-GFP/UAS-p35; FRT82B, tub-Gal80/ FRT82B or (FRT82B, *psid D4*) (panels C, G)

HsFLP; Act > y >Gal4, UAS-GFP/UAS-p35; UAS-Ubr4 RNAi,FRT82B, tub-Gal80/ FRT82B or (FRT82B, *psid D4*) (panels D-E)

HsFLP; tub-Gal80, FRT40A/ FRT40A; Act > y >Gal4, UAS-GFP / UAS-MAPK RNAi or Ubr5 RNAi (panels F, I)

HsFLP, Act>CD2>Gal4/Y; UAS-Ubr1 RNAi (panel H)

eyFLP; Act > y >Gal4, UAS-GFP/UAS-EGFR^CA^; FRT82B, tub-Gal80/ FRT82B *scrib* or (FRT82B, *psidD4*, *scrib*) (panels K, M, N)

eyFLP; Act > y >Gal4, UAS-GFP/ UAS-EGFR^CA^, UAS-psid (WT, SD, or SA); FRT82B, tub-Gal80/ FRT82B, *psidD4*, *scrib* (panel L)

## Supporting information

S1 Fig Inactivation of *rbf* and NatB subunits induce loss of double mutant tissues and synergistic cell death. (A-J) Adult eyes with single mutant clones of *psidin* alleles or double mutant clones of *psidin*, *rbf*. Mutant clones were marked by the lack of red pigment. (K-P) Large clones (white patches) of additional *psidin* alleles (K, M, and O) were observed when mutant clones were generated in a *Minute* background. In contrast, *rbf psidin* double mutant clones generated in the *Minute* background were mostly lost, resulting in smaller eyes (L, N and P). (Q-S) Cell death level (caspase 3 staining) in third instar eye/antenna discs with single or double RNAi of Rbf or NAA20. Genotype of flies used in S1 Fig: w, eyFLP /Y; FRT82B, Ubi-GFP /FRT82B (panel A), *rbf*^*15aΔ*^,w, eyFLP /Y; FRT82B, RBF-G3, Ubi-GFP/FRT82B (panel B), w, eyFLP /Y; FRT82B, Ubi-GFP /FRT82B, *psid* (*AE*,*EQ*, *psdin D1 or D4*) (panels C, E, G, I), *rbf*^*15aΔ*^,w, eyFLP /Y; FRT82B, RBF-G3, Ubi-GFP / FRT82B, *psid* (*AE*,*EQ*, *psdin D1 or D4*) (panels D, F, H, J), w, eyFLP/Y; FRT82B,*Rps3*^*-*^, Ubi-GFP/FRT82B, *psid* (*AB*, *EQ*, *or CJ*) (panels K, M, O), *rbf*^*15aΔ*^,w, eyFLP/Y; FRT82B, RBF-G3, *Rps3*^*-*^, Ubi-GFP/ FRT82B, *psid* (*AB*, *EQ*, *or CJ*) (panels L, N, P), eyFLP, Act>CD2>Gal4; UAS-Rbf RNAi / + or UAS-NAA20 RNAi (panel R-S), eyFLP, Act>CD2>Gal4; UAS-NAA20 RNAi (panel Q).
(TIF)

S2 Fig Inactivation of NatB subunits reduced EGFR signaling and slightly delayed neuronal differentiation. (A-C’) Expression level of aos-lacZ was reduced in mutant clones of additional *psidin* alleles marked by the absence of GFP. (D-F') NAA20 RNAi clones generated with EyeFlp-CoinGal4 and marked by GFP expression were stained with pErk (D-D’), and neuronal differentiation markers Elav (E and E') and Senseless (F and F'). Genotype of flies used in S2 Fig: w, eyFLP /Y; FRT82B, Ubi-GFP / aos-lacz, FRT82B, *psdin* (*AE*,*EQ*, *psid D1*) (panels A-C), eyFLP, UAS-Dcr2 / +; CoinFLP-Gal4-UAS-GFP; UAS-NAA20 RNAi (panels D-F).
(TIF)

S3 Fig Effect of *psid* mutation on activated Ras and Raf-induced MAPK activation in eye discs. (A-D) The effects of *psid-D4* mutation on activated Ras (A-B) or activated Raf (C-D) induced MAPK activation in eye discs were detected by pERK staining (red). Activated Ras-induced pERK (white arrowhead in A) was much higher than the endogenous pERK observed in the morphogenetic furrow (Yellow arrowhead in A). *psid-D4* mutation significantly reduced activated Ras-induced pERK (white arrowhead pointed in B), which was similar to the endogenous pERK level in WT tissues (yellow arrow pointed in B). Activated Raf-induced pERK (white arrow pointed in C) was not obviously affected by *psid-D4* mutation (white arrow pointed in D). Genotype of flies used in S3 Fig: HsFLP; Act > y >Gal4, UAS-GFP/UAS-Ras^v12^; FRT82B, tub-Gal80/ FRT82B or (FRT82B, *psidD4*) (panels A-B), eyFLP; Act > y >Gal4, UAS-GFP/UAS-Raf^gof^; FRT82B, tub-Gal80/ FRT82B or (FRT82B, *psidD4*) (panels C-D).
(TIF)

S4 Fig Knockdown NAA20 did not affect Drk mRNA level and Knockdown E3 ubiquitin ligase Ubr1 did not restore Drk protein level in *psid* mutant clones. (A) mRNA levels from eye antenna discs expressing LexA control RNAi or NAA20-RNAi were determined by qRT-PCR. # indicates no significant statistical difference. (B) *psid-D1* MARCM clones in wing discs, marked by GFP expression (pointed by arrowheads), showed decreased Drk level. (C) Ubr1 RNAi did not rescue the decreased Drk level in *psid-D1* clones marked by GFP expression (pointed by arrowheads). Genotype of flies used in S4 Fig: eyFLP; Act > y >Gal4, UAS-LexA-RNAi (or NAA20-RNAi) (panel A), HsFLP; Act > y >Gal4, UAS-GFP; FRT82B, tub-Gal80/ FRT82B, *psid D1 or* (*UAS-Ubr1 RNAi*, FRT82B, *psid D1*) (panels B-C).
(TIF)

S5 Fig PP2AC mRNA level was not affected by knockdown NAA20 or Cnot4 and PP2AC protein level was not affected by inactivation of E3 ubiquitin ligases CG1317/Teb4, Ubr1, or Ubr5. (A) Genomic structure of a deletion allele of *CG1317/Teb4 Δ6–1*, generated from imprecise excision of *CG1317 P* element (BL20646). DNA sequencing data revealed a 2078 bp deletion, which starts from 14 bp upstream of CG1317. The numbers in the diagram indicate the precise location of deletion in the fly genome. (B) *CG1317/Teb4 Δ6–1* mutant clones (pointed by white arrowhead) in eye disc, marked by lack of GFP, did not affect PP2AC levels. (C) Quantitative RT-PCR results of RNA isolated from 3^rd^ instar eye/antenna discs expressing LexA control RNAi and NAA20 RNAi, or from Cnot4 RNAi and control W RNAi expressing. # indicates no significant difference was observed in PP2AC mRNA levels. (D-E) Clones of cells (pointed by white arrowheads) with GFP and Ubr1 RNAi (D) or Ubr5 RNAi (E) expression did not affect PP2AC levels in eye discs. (F) *CG1317/Teb4 Δ6–1* mutant clones (pointed by white arrowhead) in eye disc, marked by lack of GFP, did not affect Drk levels. Genotype of flies used in S5 Fig: w, eyFLP; Ubi-GFP, FRT80B/ *Teb4*, FRT80B (panel B, F), eyFLP; Act > y >Gal4, UAS-LexA-RNAi (or NAA20-RNAi, W-RNAi, Cnot4-RNAi) (panel C), HsFLP; tub-Gal80, FRT40A/ FRT40A; Act > y >Gal4, UAS-GFP / UAS-Ubr5 RNAi (panel E), HsFLP, Act>CD2>Gal4/Y; UAS-Ubr1 RNAi (panel D).
(TIF)

S6 Fig Effect of *psid* mutation on Sprouty protein level. Reduced levels of Spry were observed in clones of cells expressing Spry RNAi (A, RNAi cells were labeled with GFP). White and yellow arrowheads in (A) point to RNAi cells located in the posterior or the anterior region of eye disc. Reduced levels of Spry were also observed in *psid-D1* mutant clones (B, white arrowhead. Mutant clones were marked by absence of GFP). (C-D) expression of HA tagged Spry (shown in red) with GFP (shown in green) in control (C-C”) or *psid* mutant (D-D”) MARCM clones. (E) Spry-HA levels normalized by GFP signal in FRT control or *psid* mutant clones were shown. (F-G’) Images of wing discs with PTP-ER-RNAi flip-out clones (shown in green, pointed by yellow arrowheads) stained by two PTP-ER monoclonal antibodies (26E4C7 and 2D7F8). Genotype of flies used in S6 Fig: eyFLP, UAS-Dcr2 / +; CoinFLP-Gal4-UAS-GFP; UAS-Sprouty RNAi (panel A), HsFLP; FRT82B,Ubi-GFP / FRT82B, *psid D1* (panel B), HsFLP; Act > y >Gal4, UAS-GFP / UAS-Spry; FRT82B, tub-Gal80/ FRT82B or (FRT82B, *psid D4*) (panel C-D).
(TIF)

S7 Fig Proposed model for the regulation of EGFR signaling by NatB Nt-acetylation and the two branches of N-end rule pathways. (A) when NatB is inhibited or when Ac-CoA level is low, most Drk, MAPK and PP2AC will not be Nt-acetylated, which results in the inhibition of EGFR signaling due to the accumulation of PP2AC, a negative component of the pathway, and the loss of Drk and MAPK, the positive components of the pathway. (B) When NatB activity is high or when Ac-CoA level is high, more Drk, MAPK, and PP2AC will be Nt-acetylated, which results in the higher levels of EGFR signaling due to the accumulation of acetylated Drk and MAPK and the loss of PP2AC.
(TIF)
