# Supplementary figures and images for: NatB regulates Rb mutant cell death and tumor growth by modulating EGFR/MAPK signaling through the N-end rule pathways
Source: PLoS Genet. 2020 Jun 19;16(6):e1008863. doi: 10.1371/journal.pgen.1008863 (PMC7329143; doi:10.1371/journal.pgen.1008863)

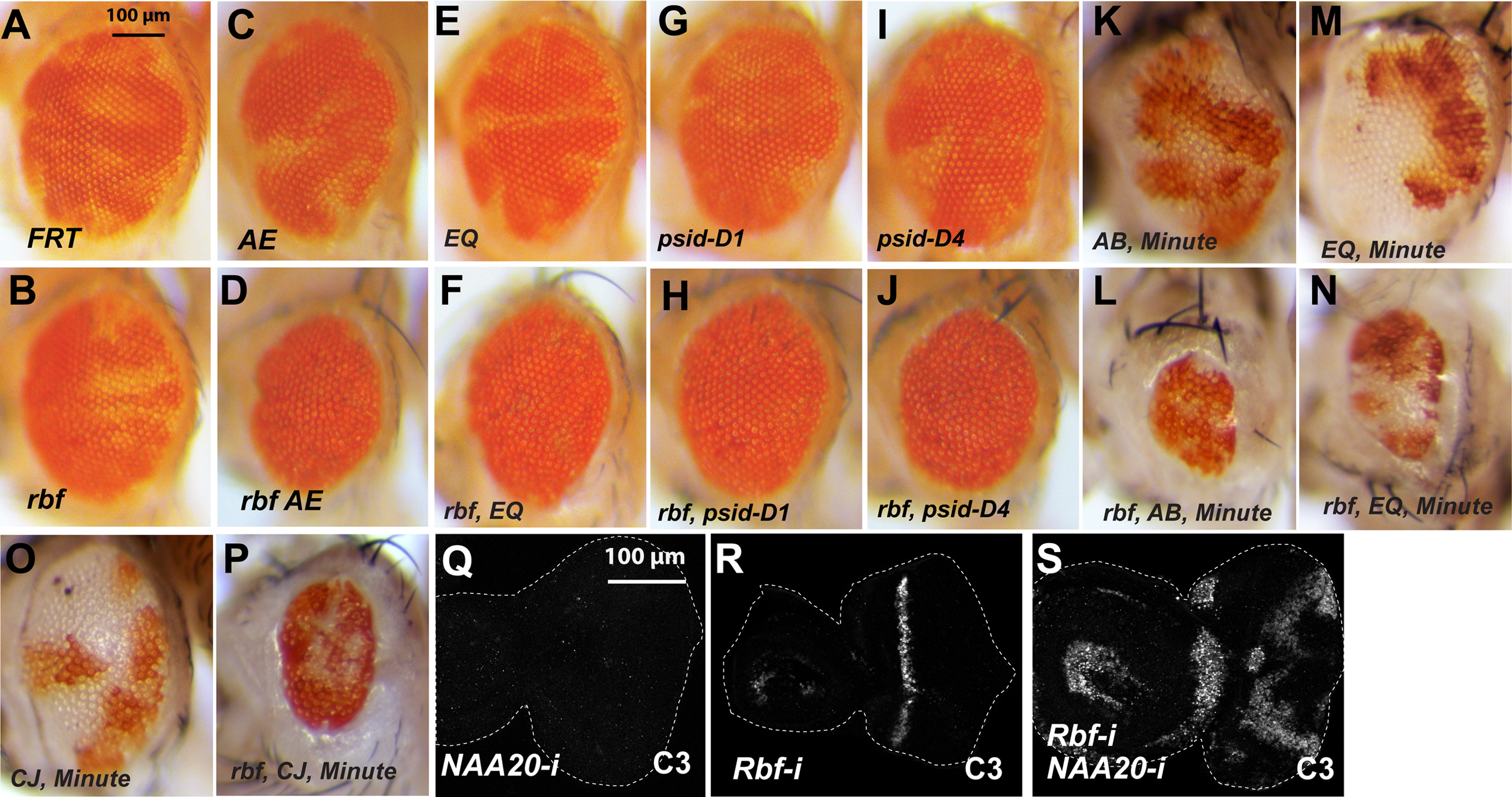

Supplement: S1 Fig — (A-J) Adult eyes with single mutant clones of psidin alleles or double mutant clones of psidin, rbf. Mutant clones were marked by the lack of red pigment. (K-P) Large clones (white patches) of additional psidin alleles (K, M, and O) were observed when mutant clones were generated in a Minute background. In contrast, rbf psidin double mutant clones generated in the Minute background were mostly lost, resulting in smaller eyes (L, N and P). (Q-S) Cell death level (caspase 3 staining) in third instar eye/antenna discs with single or double RNAi of Rbf or NAA20. Genotype of flies used in S1 Fig: w, eyFLP /Y; FRT82B, Ubi-GFP /FRT82B (panel A), rbf15aΔ,w, eyFLP /Y; FRT82B, RBF-G3, Ubi-GFP/FRT82B (panel B), w, eyFLP /Y; FRT82B, Ubi-GFP /FRT82B, psid (AE,EQ, psdin D1 or D4) (panels C, E, G, I), rbf15aΔ,w, eyFLP /Y; FRT82B, RBF-G3, Ubi-GFP / FRT82B, psid (AE,EQ, psdin D1 or D4) (panels D, F, H, J), w, eyFLP/Y; FRT82B,Rps3-, Ubi-GFP/FRT82B, psid (AB, EQ, or CJ) (panels K, M, O), rbf15aΔ,w, eyFLP/Y; FRT82B, RBF-G3, Rps3-, Ubi-GFP/ FRT82B, psid (AB, EQ, or CJ) (panels L, N, P), eyFLP, Act>CD2>Gal4; UAS-Rbf RNAi / + or UAS-NAA20 RNAi (panel R-S), eyFLP, Act>CD2>Gal4; UAS-NAA20 RNAi (panel Q). (TIF) [file pgen.1008863.s001.tif]

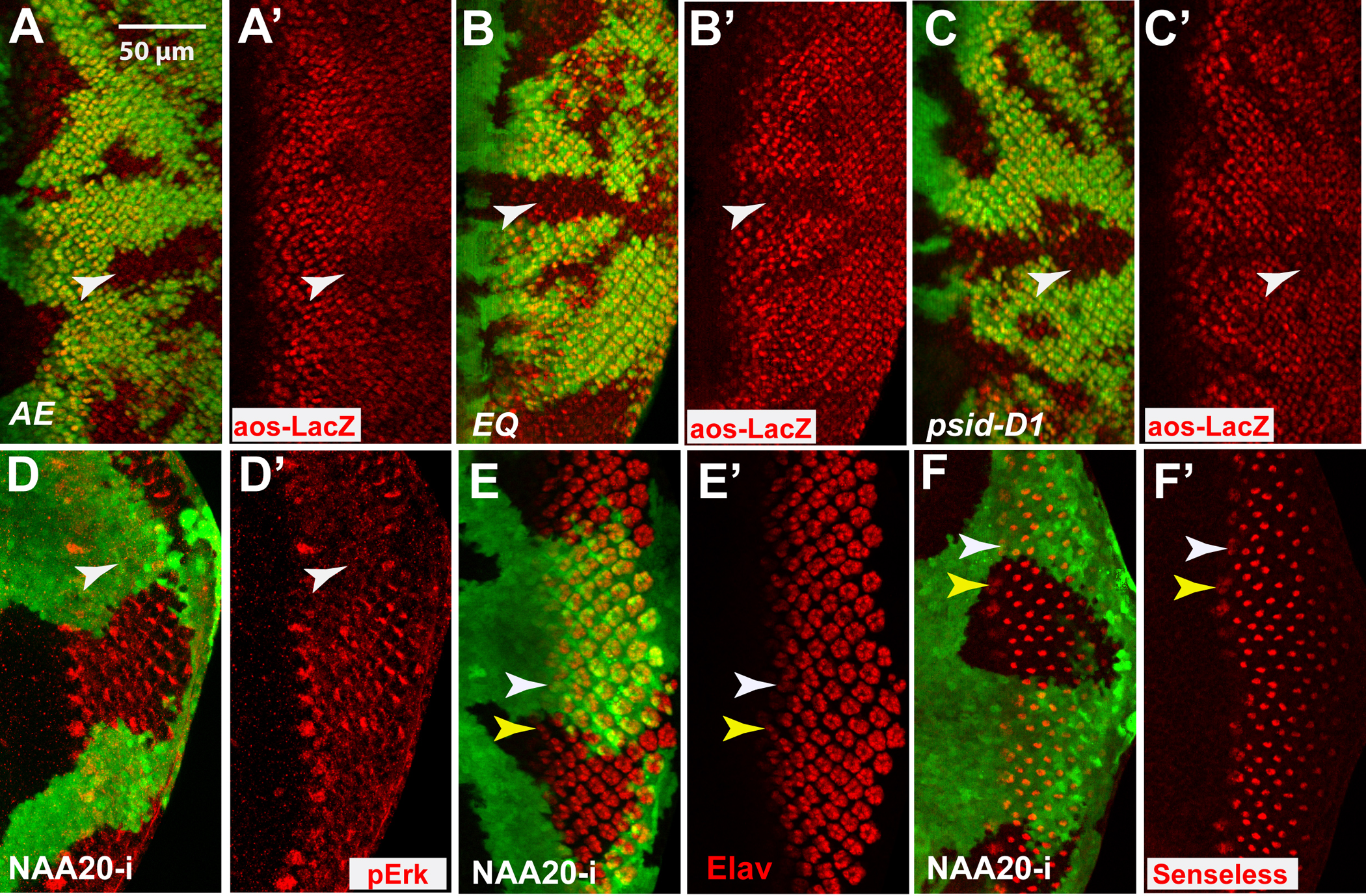

Supplement: S2 Fig — (A-C’) Expression level of aos-lacZ was reduced in mutant clones of additional psidin alleles marked by the absence of GFP. (D-F') NAA20 RNAi clones generated with EyeFlp-CoinGal4 and marked by GFP expression were stained with pErk (D-D’), and neuronal differentiation markers Elav (E and E') and Senseless (F and F'). Genotype of flies used in S2 Fig: w, eyFLP /Y; FRT82B, Ubi-GFP / aos-lacz, FRT82B, psdin (AE,EQ, psid D1) (panels A-C), eyFLP, UAS-Dcr2 / +; CoinFLP-Gal4-UAS-GFP; UAS-NAA20 RNAi (panels D-F). (TIF) [file pgen.1008863.s002.tif]

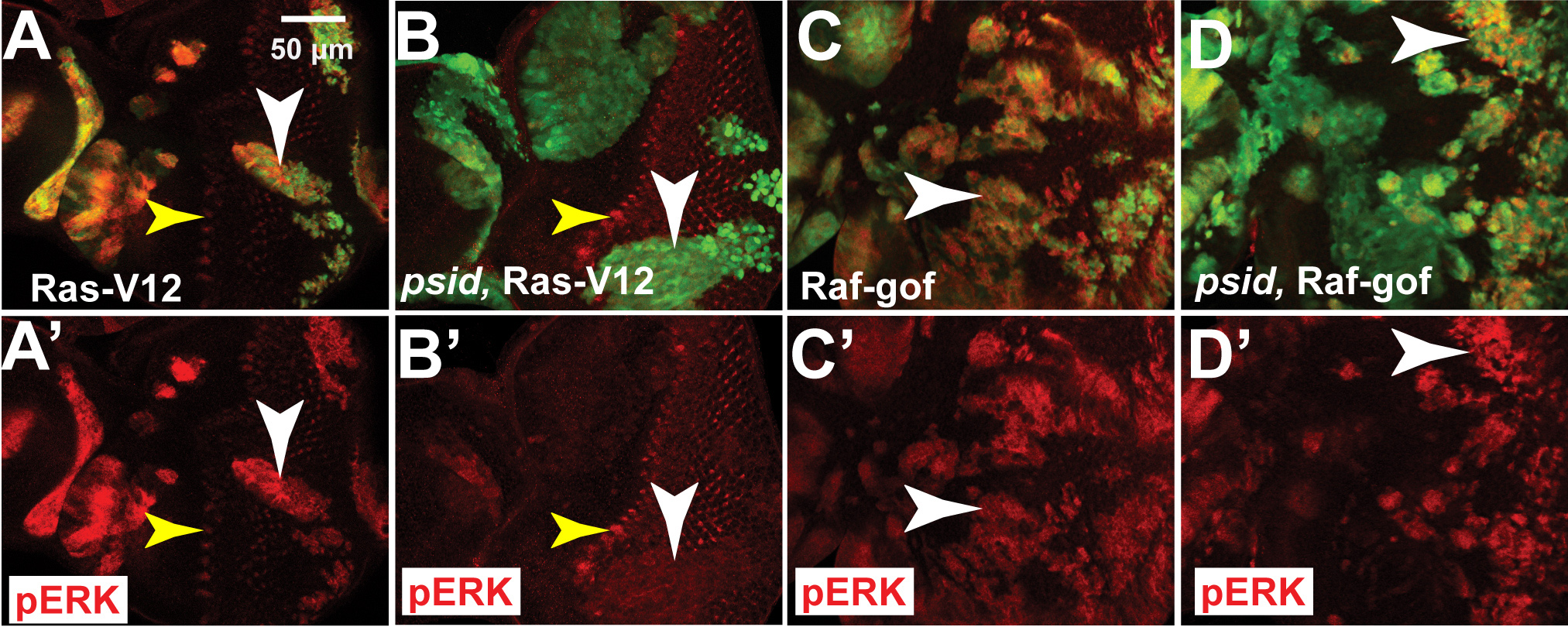

Supplement: S3 Fig — (A-D) The effects of psid-D4 mutation on activated Ras (A-B) or activated Raf (C-D) induced MAPK activation in eye discs were detected by pERK staining (red). Activated Ras-induced pERK (white arrowhead in A) was much higher than the endogenous pERK observed in the morphogenetic furrow (Yellow arrowhead in A). psid-D4 mutation significantly reduced activated Ras-induced pERK (white arrowhead pointed in B), which was similar to the endogenous pERK level in WT tissues (yellow arrow pointed in B). Activated Raf-induced pERK (white arrow pointed in C) was not obviously affected by psid-D4 mutation (white arrow pointed in D). Genotype of flies used in S3 Fig: HsFLP; Act > y >Gal4, UAS-GFP/UAS-Rasv12; FRT82B, tub-Gal80/ FRT82B or (FRT82B, psidD4) (panels A-B), eyFLP; Act > y >Gal4, UAS-GFP/UAS-Rafgof; FRT82B, tub-Gal80/ FRT82B or (FRT82B, psidD4) (panels C-D). (TIF) [file pgen.1008863.s003.tif]

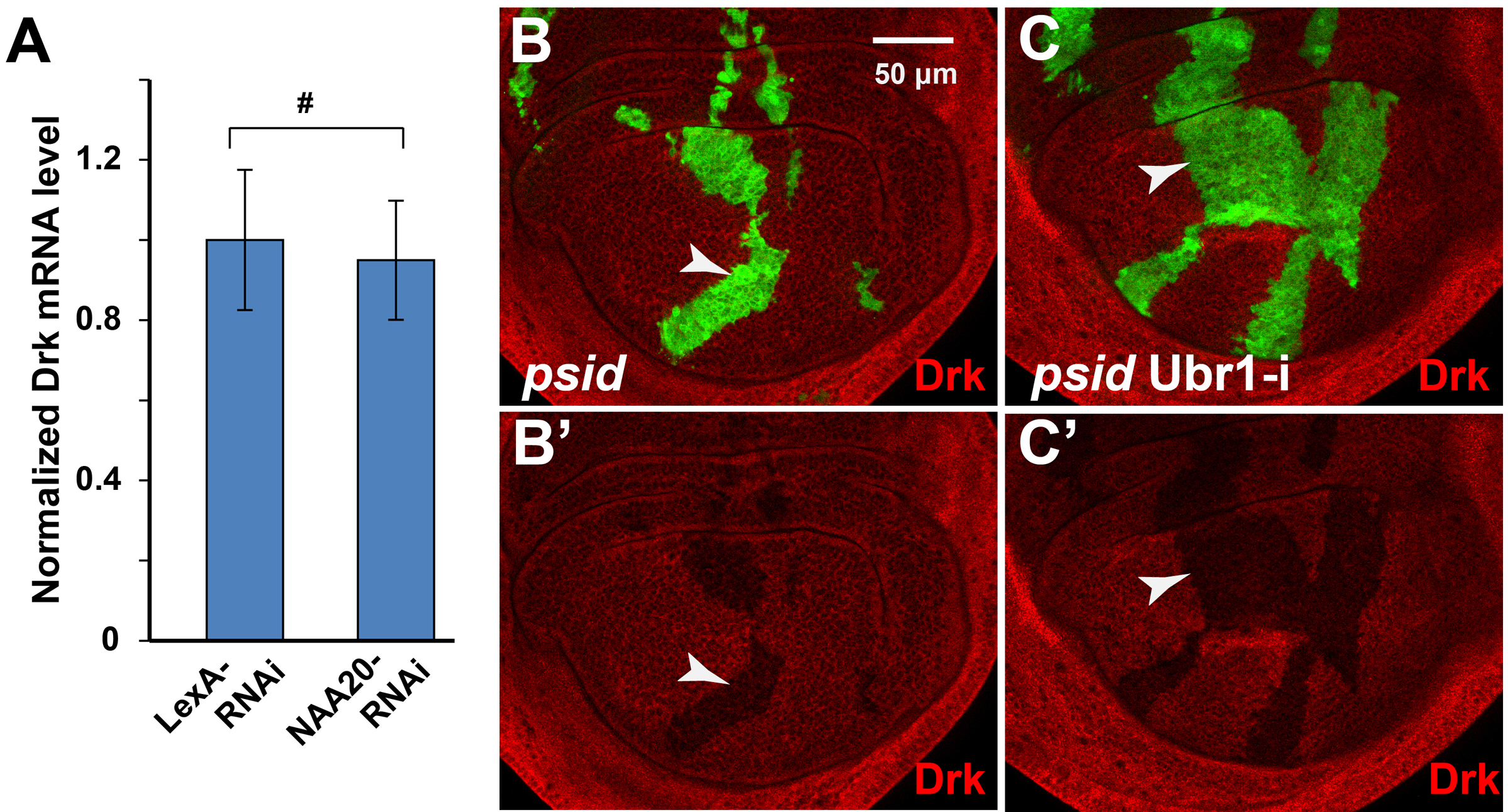

Supplement: S4 Fig — (A) mRNA levels from eye antenna discs expressing LexA control RNAi or NAA20-RNAi were determined by qRT-PCR. # indicates no significant statistical difference. (B) psid-D1 MARCM clones in wing discs, marked by GFP expression (pointed by arrowheads), showed decreased Drk level. (C) Ubr1 RNAi did not rescue the decreased Drk level in psid-D1 clones marked by GFP expression (pointed by arrowheads). Genotype of flies used in S4 Fig: eyFLP; Act > y >Gal4, UAS-LexA-RNAi (or NAA20-RNAi) (panel A), HsFLP; Act > y >Gal4, UAS-GFP; FRT82B, tub-Gal80/ FRT82B, psid D1 or (UAS-Ubr1 RNAi, FRT82B, psid D1) (panels B-C). (TIF) [file pgen.1008863.s004.tif]

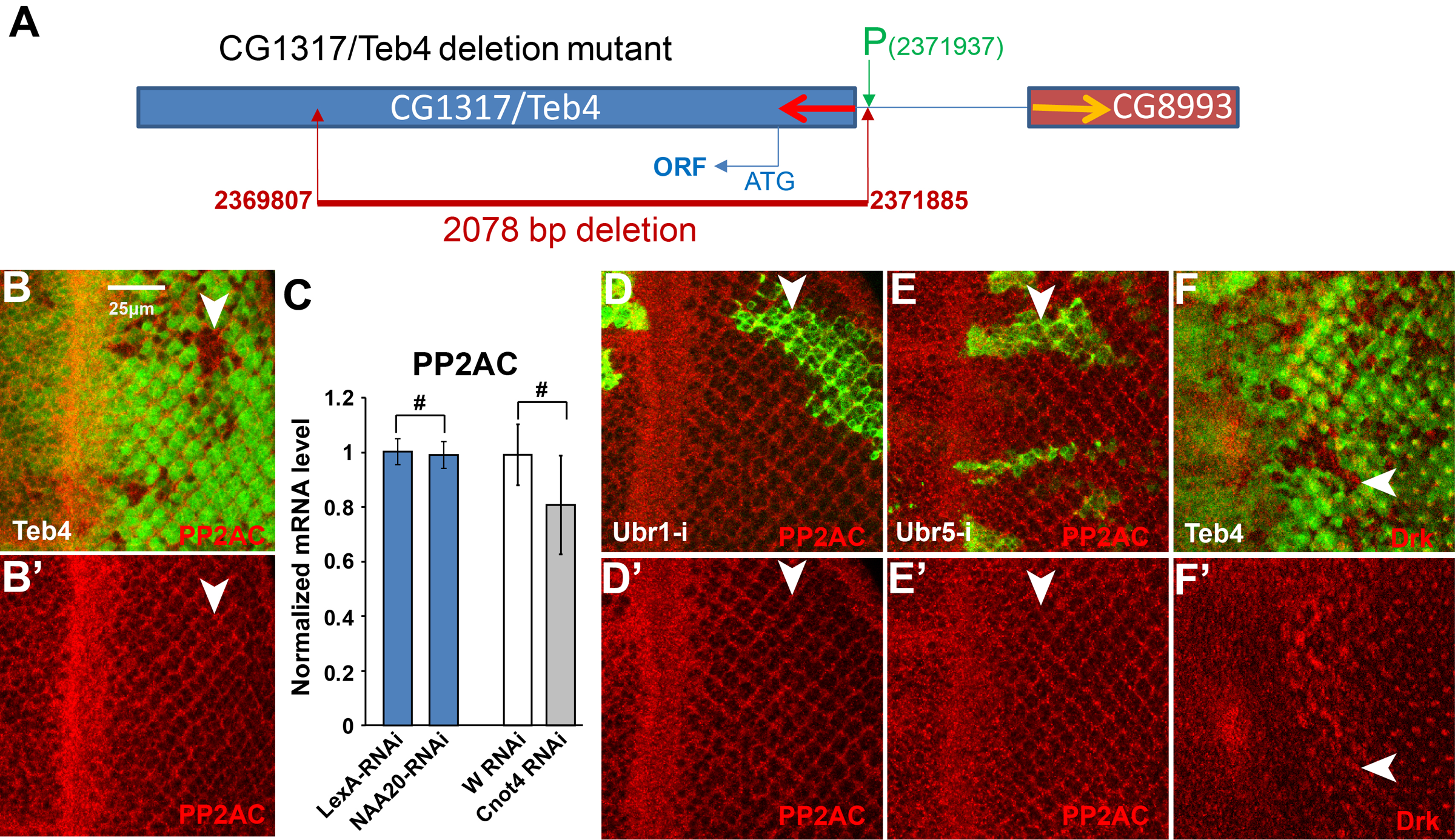

Supplement: S5 Fig — (A) Genomic structure of a deletion allele of CG1317/Teb4 Δ6–1, generated from imprecise excision of CG1317 P element (BL20646). DNA sequencing data revealed a 2078 bp deletion, which starts from 14 bp upstream of CG1317. The numbers in the diagram indicate the precise location of deletion in the fly genome. (B) CG1317/Teb4 Δ6–1 mutant clones (pointed by white arrowhead) in eye disc, marked by lack of GFP, did not affect PP2AC levels. (C) Quantitative RT-PCR results of RNA isolated from 3rd instar eye/antenna discs expressing LexA control RNAi and NAA20 RNAi, or from Cnot4 RNAi and control W RNAi expressing. # indicates no significant difference was observed in PP2AC mRNA levels. (D-E) Clones of cells (pointed by white arrowheads) with GFP and Ubr1 RNAi (D) or Ubr5 RNAi (E) expression did not affect PP2AC levels in eye discs. (F) CG1317/Teb4 Δ6–1 mutant clones (pointed by white arrowhead) in eye disc, marked by lack of GFP, did not affect Drk levels. Genotype of flies used in S5 Fig: w, eyFLP; Ubi-GFP, FRT80B/ Teb4, FRT80B (panel B, F), eyFLP; Act > y >Gal4, UAS-LexA-RNAi (or NAA20-RNAi, W-RNAi, Cnot4-RNAi) (panel C), HsFLP; tub-Gal80, FRT40A/ FRT40A; Act > y >Gal4, UAS-GFP / UAS-Ubr5 RNAi (panel E), HsFLP, Act>CD2>Gal4/Y; UAS-Ubr1 RNAi (panel D). (TIF) [file pgen.1008863.s005.tif]

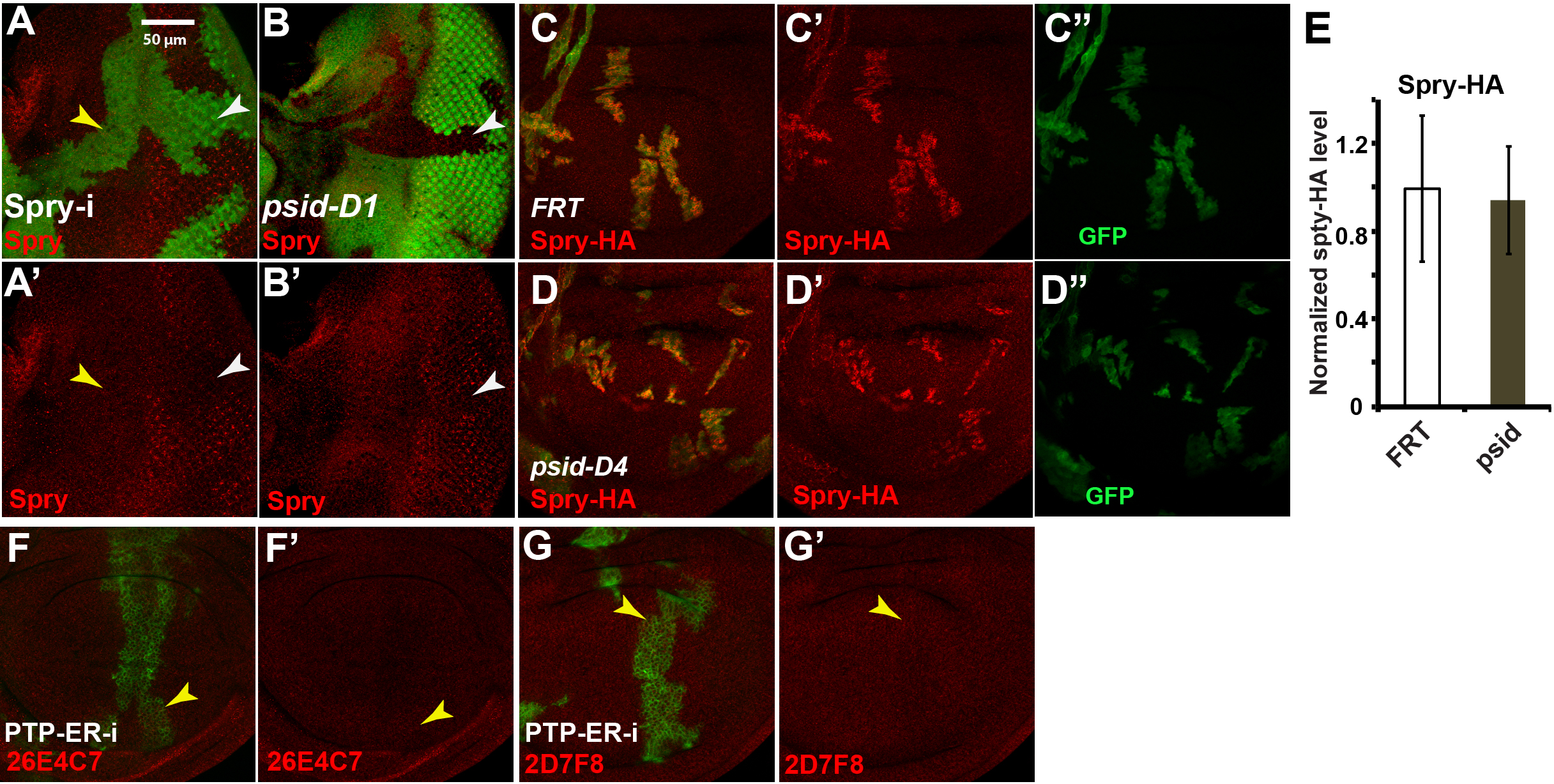

Supplement: S6 Fig — Reduced levels of Spry were observed in clones of cells expressing Spry RNAi (A, RNAi cells were labeled with GFP). White and yellow arrowheads in (A) point to RNAi cells located in the posterior or the anterior region of eye disc. Reduced levels of Spry were also observed in psid-D1 mutant clones (B, white arrowhead. Mutant clones were marked by absence of GFP). (C-D) expression of HA tagged Spry (shown in red) with GFP (shown in green) in control (C-C”) or psid mutant (D-D”) MARCM clones. (E) Spry-HA levels normalized by GFP signal in FRT control or psid mutant clones were shown. (F-G’) Images of wing discs with PTP-ER-RNAi flip-out clones (shown in green, pointed by yellow arrowheads) stained by two PTP-ER monoclonal antibodies (26E4C7 and 2D7F8). Genotype of flies used in S6 Fig: eyFLP, UAS-Dcr2 / +; CoinFLP-Gal4-UAS-GFP; UAS-Sprouty RNAi (panel A), HsFLP; FRT82B,Ubi-GFP / FRT82B, psid D1 (panel B), HsFLP; Act > y >Gal4, UAS-GFP / UAS-Spry; FRT82B, tub-Gal80/ FRT82B or (FRT82B, psid D4) (panel C-D). (TIF) [file pgen.1008863.s006.tif]

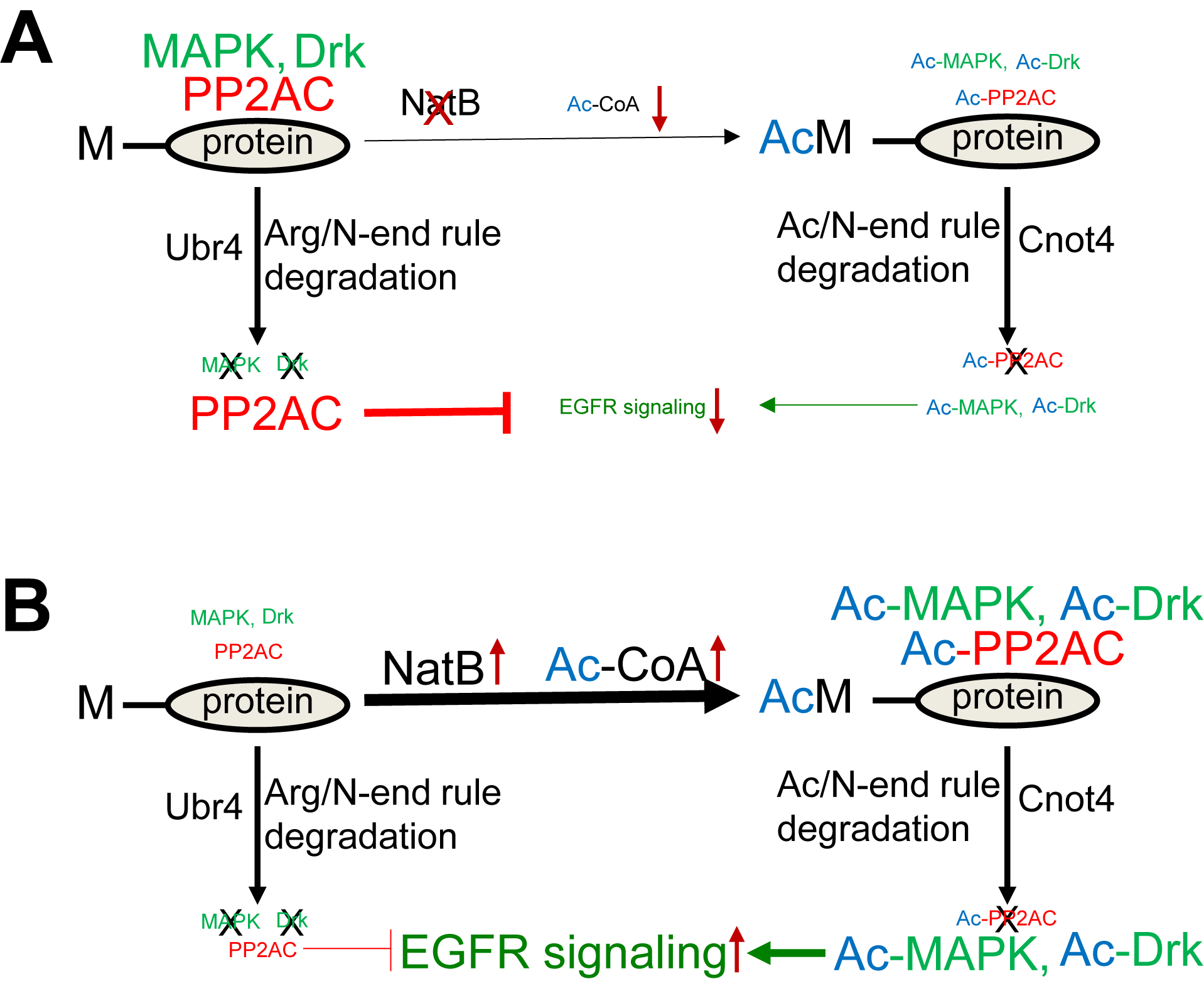

Supplement: S7 Fig — (A) when NatB is inhibited or when Ac-CoA level is low, most Drk, MAPK and PP2AC will not be Nt-acetylated, which results in the inhibition of EGFR signaling due to the accumulation of PP2AC, a negative component of the pathway, and the loss of Drk and MAPK, the positive components of the pathway. (B) When NatB activity is high or when Ac-CoA level is high, more Drk, MAPK, and PP2AC will be Nt-acetylated, which results in the higher levels of EGFR signaling due to the accumulation of acetylated Drk and MAPK and the loss of PP2AC. (TIF) [file pgen.1008863.s007.tif]
